# Magic roundabout is an endothelial-specific ohnolog of ROBO1 which neo-functionalized to an essential new role in angiogenesis

**DOI:** 10.1371/journal.pone.0208952

**Published:** 2019-02-25

**Authors:** Lukasz Huminiecki

**Affiliations:** Instytut Genetyki i Hodowli Zwierząt Polskiej Akademii Nauk, Jastrzębiec, Magdalenka, Poland; Yale University School of Medicine, UNITED STATES

## Abstract

**Background:**

Magic roundabout (ROBO4) is an unusual endothelial-specific paralog of the family of neuronally-expressed axon guidance receptors called roundabouts. Endothelial cells (ECs), whose uninterrupted sheet delimits the lumen of all vertebrate blood vessels and which are absent from invertebrate species, are a vertebrate-specific evolutionary novelty.

**Results:**

Herein, the evolutionary mechanism of the duplication, retention and divergence of ROBO4 was investigated for the first time. Phylogenetic analyses carried out suggested that ROBO4 is a fast-evolving paralog of ROBO1 formed at the base of vertebrates. The ancestral expression pattern was neuronal. ROBO4 dramatically shifted its expression and became exceptionally specific to ECs. The data-mining of FANTOM5 and ENCODE reveals that ROBO4’s endothelial expression arises from a single transcription start site (TSS), conserved in mouse, controlled by a proximal promoter with a complex architecture suggestive of regulatory neo-functionalization. (An analysis of promoter probabilities suggested the architecture was not due to a chance arrangement of TFBSes). Further evidence for the neo-functionalization of ROBO4 comes from the analysis of its protein interactions, the rates of protein evolution, and of positively selected sites.

**Conclusions:**

The neo-functionalization model explains why ROBO4 protein acquired new context-specific biological functions in the control of angiogenesis. This endothelial-specific roundabout receptor is an illustrative example of the emergence of an essential vertebrate molecular novelty and an endothelial-specific signaling sub-network through 2R-WGD. The emergence of novel cell types, such as ECs, might be a neglected evolutionary force contributing to the high rate of retention of duplicates post-2R-WGD. Crucially, expression neo-functionalization to evolutionarily novel sites of expression conceptually extends the classical model of neo-functionalization.

## Introduction

An endothelial-specific paralog of an axon guidance receptor ROBO1 was cloned and called magic roundabout, or ROBO4 [1]. ROBO4 was identified, among four other novel endothelial-specific genes, in a two-thronged bioinformatics data-mining procedure. The procedure combined data from two technological platforms for expression profiling: expressed sequence tags and serial analysis of gene expression. Both these expression profiling technologies were allowed to vote for a consensus set of candidate endothelial-specific molecules which were verified experimentally with RT-PCR.

Two striking features of ROBO4 distinguished it from three other roundabouts (ROBO1-3) in the human genome: that it was endothelial-specific in its expression, and that it was diverged in sequence and lacked some of the extra-cellular domains typical of roundabouts [2]. It was intriguing that an endothelial-specific member of a family recognized mostly for its neuronal expression and the function in axon guidance [3, 4] was cloned. The molecule has attracted considerable attention both in academia and in industry. When on 30th June 2017 MEDLINE was searched for ROBO4, 122 publications were found. However, it is still uncertain why the expression pattern of ROBO4, as well as its protein sequence, are so profoundly diverged.

Despite the intense interest in ROBO4 among molecular and vascular biologists as well as among molecular oncologists, an analysis of evolutionary forces responsible for the retention of the ROBO4 paralog has not been published. Thus, it has been unclear which model of the evolution of gene duplicates actually explains ROBO4. This knowledge gap should be urgently filled also because there is much interest in the evolutionary origins of the endothelium which is absent from invertebrate circulatory systems but present in every vertebrate without exception [5]. The endothelium consist of a single layer of endothelial cells (ECs) which delimit the lumen of the entire vertebrate vascular system. In vertebrates, the ECs are the main instrument of angiogenesis—the process of the formation of new blood vessels from pre-existing vasculature [6].

As a general rule, animal gene families, such as roundabouts, emerge through consecutive rounds of gene duplications. (Horizontal gene transfer is thought of as rare in animals). Gene duplications occur all the time for all loci, at an estimated average of 0.01 per gene in a million years [7]. However, only a minority of duplicates are retained [8]. Originally, neo-functionalization was proposed to be the main force behind the retention of duplicates [9]. In this model, a duplicated gene acquires a new and essential function that is beneficial for the organism. Subsequently, the evolutionary forces of purifying selection act to retain the neo-functionalized copy of the ancestral gene, as well as the copy carrying on the ancestral function (as both are now indispensable). Originally such new functions were thought of in terms of the mutations affecting protein coding sequences and leading to a change in biochemical activities [8, 10]. However, there has been a growing realization that in multicellular organisms neo-functionalization can be also thought of in terms of acquiring news sites of expression [11–13]. That is to say, a gene can neo-functionalize to become expressed in a cell type or a tissue type in which the ancestral gene was not previously transcribed. In case of vertebrates, a given cell- or tissue-type might have not existed in the ancestral pre-2R organism, entangling the emergence of novel genes with the emergence of entirely novel cell types with novel transcriptional regulatory networks.

Conant and Wolfe [14] underlined that an early expression shift may set the stage for a subsequent evolution of a new protein function. These authors also discussed models alternative to neo-functionalization such as duplication degeneration complementation [15], or the DDC model, and the escape from adaptive conflict [16–18], or the EAC model. DDC was introduced to explain the high rate of duplicate retention specifically following animal WGDs. Crucially, the DDC model makes testable predictions about promoter architectures of paralogs preserved by regulatory sub-functionalization. What matters most for this analysis is that under the DDC model paralogs ought to differentially preserve regulatory modules of the ancestral locus. In stark contrast, regulatory neo-functionalization ought to proceed via the formation of novel promoter architectures, which might be acquired entirely *de novo* or fortuitously during duplication.

What is the molecular mechanism of duplication? Most gene duplications are tandem duplications which lead to the formation of collinear chromosomal clusters. However, whole genome duplication (WGD) duplicates all genes in the genome simultaneously through polyploidisation. WGDs are rare in animals, but two rounds of WGD (2R-WGD) occurred at the base of vertebrates [9, 19, 20] leading to the formation of many vertebrate evolutionary novelties [21]. Results from plants [22, 23], animals [21], and yeasts [24] independently suggested that WGDs lead to preferential retention of transcription factors (TFs) and genes involved in signal transduction which are generally not duplicated through single-gene duplications. The mechanism of this is thought to be that single-gene duplications of proteins involved in complexes of dosage-sensitive proteins are harmful [25, 26].

While we can be certain of the general association between genes retained in duplicated copies after 2R-WGD and signal transduction, there is still a great need to understand in detail how exactly individual genes, such as ROBO4, found their new roles. Distinguishing between neo-functionalization and alternative mechanisms, as well as between regulatory vs. protein-level evolution, is critical. It is also important to retrace how promoters of paralogs diverge, and how cellular networks were structurally or functionally affected by new ohnologs and the emergence of novel cell types. This may require some detective work and detailed analyses of individual gene families. The aim of this study is to perform such an analysis for roundabouts.

Modern functional genomics resources such as ENCODE and FANTOM5 (F5) can help us be such Darwinian molecular detectives. ENCODE was an international project aiming at the characterization of functional elements in the human genome and includes ChIP-seq data for 161 TFs [27]. ENCODE’s data can help to define the architectures of promoters, that is the sets of TFs which they can bind [28], and to distinguish between regulatory neo- and sub-functionalization. Crucially, ENCODE’s are experimentally-defined protein-DNA binding events: a major advantage over *de novo* computational predictions available before. It is possible to detect motifs *de novo* but motif accessibility is a crucial factor in the regulation of mammalian transcription. Detailed examination of individual gene families can, in turn, help interpret ENCODE data. For example, the functional significance of frequent low-affinity low-occupancy binding sites of TFs (so called weak TFBSes) has been subject to much discussion and occasionally heated controversy [29–31]. Moreover, it is yet unclear how empirical ENCODE data will impact the theoretical models of neo- and sub-functionalization which might need updating in the era of experimentally-defined promoter architectures [28].

The second modern functional genomics resource used here, F5, was the fifth edition of the Functional Annotation of the Mammalian Genome Consortium [32]. F5 generated a comprehensive atlas of vertebrate expression profiles (with the focus on human and mouse) at a single-base resolution. The consortium used the cutting-edge technology of the cap analysis of gene expression (CAGE) which, unlike microarrays, scans the entire genome in an unbiased fashion. Unlike RNAseq, CAGE enables the discovery of alternative TSSes, putting the spotlight on the evolution of gene expression on the level of individual TSSes, and urging us to update the models of duplicate retention. Again, the flow of information and interpretation can be bi-directional. Detailed examination of individual gene families and loci can help determine which of F5’s many alternative TSSes are functional, which are conserved, and what promoter architectures and DNA binding motifs they are linked to.

**Fig 1:**
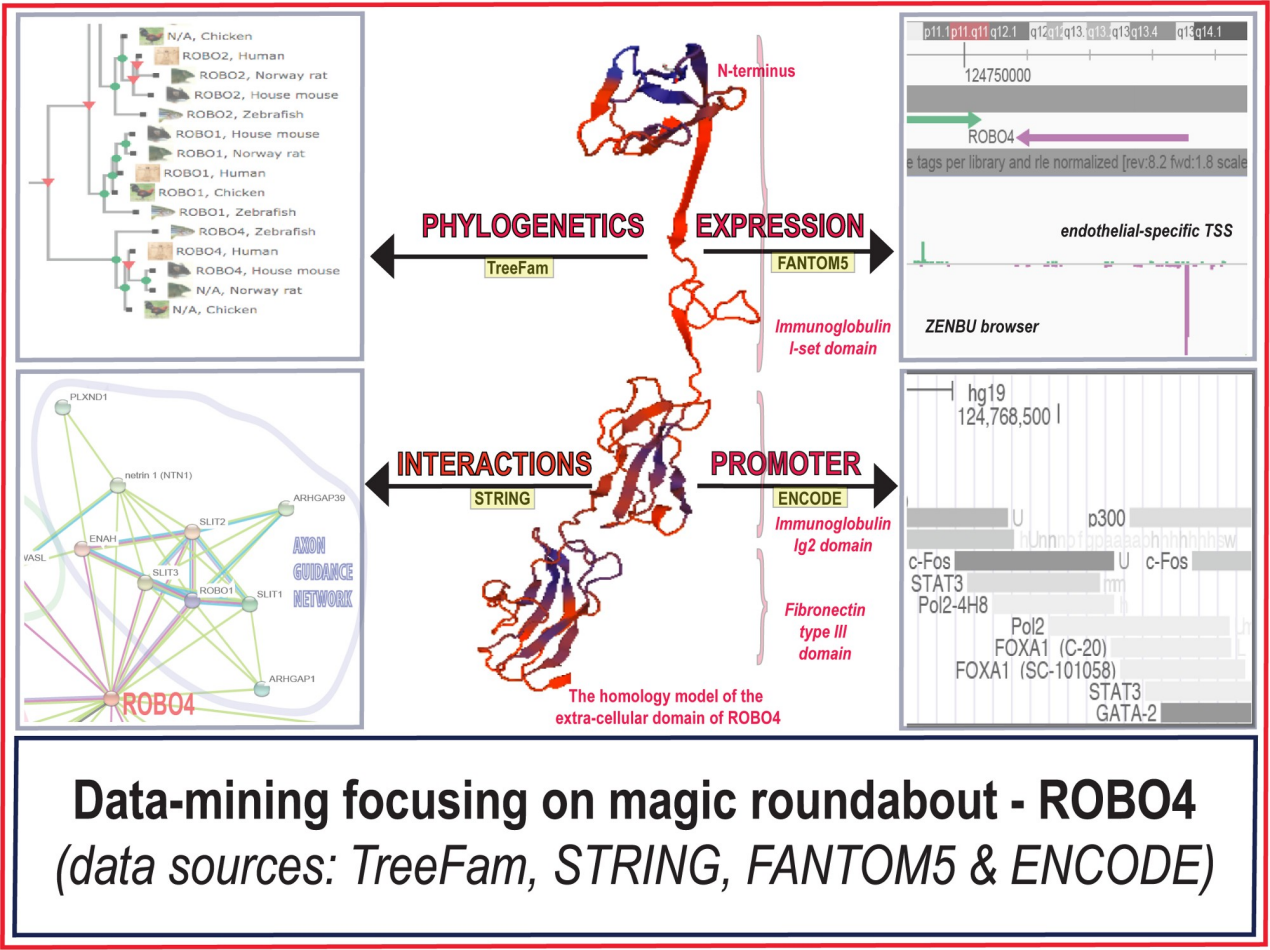
The directions of the data-mining of the ROBO4 paralog. This conceptual figure illustrates the four major directions of this analysis. These include: (1) the phylogenetics of roundabouts, (2) the protein interactions of ROBO4, (3) the gene expression patterns of roundabouts, and (4) the architecture of the promoter of ROBO4. In the center of the figure, ROBO4 is symbolized by the homology model of the extra-cellular domain of the gene. (Note that there is yet no crystal structure of ROBO4.).

Herein, we set out to test whether the emergence of ROBO4 can be explained by the evolutionary model of neo-functionalization. The answer was overwhelmingly positive. In the conceptual figure (Fig 1), the following four direction of the analysis are outlined: (1) a phylogenetic tree; (2) the protein interaction network; (3) expression patterns and alternative TSSes; and (4) promoter architectures. Additionally, the protein sequence of ROBO4 was tested for the signal of positive selection. The results are discussed in the context of more general contributions of 2R-WGD to the emergence of vertebrate vascular endothelium. We also speculate that the emergence of novel cell types could increase the frequency of neo-functionalization.

## Results

### The phylogenetic history of roundabouts

**Fig 2:**
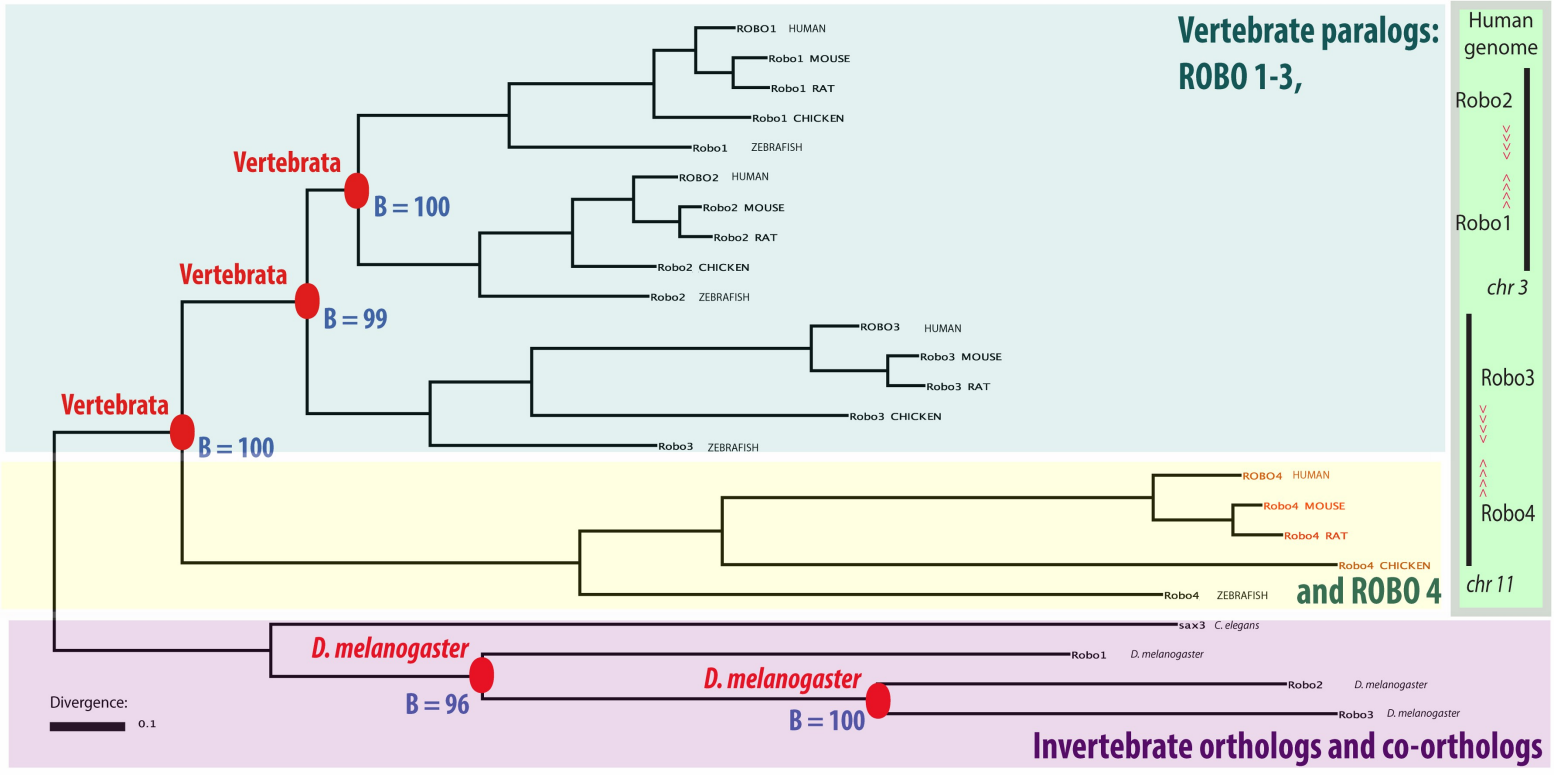
The phylogenetic tree of roundabouts. In the tree, nodes corresponding to gene duplications were annotated with a bootstrap value (B) and the taxon of duplication (note red labels). The tree suggests a single ancestral bilaterian roundabout in the last common ancestor of vertebrates and insects. There are four vertebrate roundabout paralogs: ROBO1, ROBO2, ROBO3 and ROBO4. They derive from gene duplications timed by phylogenetic timing to the base of vertebrates. In addition, in *D*. *melanogaster* there were two lineage-specific duplications giving rise to three paralogs: robo1-3 (but these duplications were not the focus of our analyses). The tree was calculated from the protein-guided nucleotide alignment of roundabout sequences, displayed using TreeViewJ [102], and annotated graphically in Adobe Illustrator using data from the .nhx tree file (S5 File). Extant species are signified with the following labels: HUMAN—*Homo sapiens*, MOUSE—*Mus musculus*, RAT—*Rattus Norvegicus*, CHICKEN—, *Gallus gallus*, BRARE—*Danio rerio*, CAEEL—*Caenorhabditis elegans*, DROME—*Drosophila melanogaster*. The tree is rooted on time.

We started by constructing a phylogenetic tree of roundabouts which is shown in Fig 2. The tree was computed using the TreeBest hybrid tree builder [33]. This methodological choice is justified by the fact that TreeBeST previously performed well for the analysis of TGF-β receptors and SMADs [34], and other signaling pathways [21], which evolved according to the 2R model. The method timed duplications in a manner consistent with the knowledge of experts in the field of TGF-β signaling.

Here, one ought to note that roundabouts exist only in bilaterian animals. (The same is true of confirmed and putative ligands of roundabouts: Slits and netrin receptors—data not shown.) In vertebrates, there are four roundabout paralogs: ROBO1-4 which derive from duplications dated to the base of vertebrates. Such duplications were linked with 2R-WGD and are enriched in signaling genes [21]. Note that ROBO1-4 are exactly of the same evolutionary age yet they differ greatly in the lengths of their branches. (The branch of ROBO4 is the longest, that of ROBO3 is the second longest). This suggests differences in the evolutionary rates of divergence. A single roundabout receptor can be inferred in the last common ancestor of vertebrates and invertebrates. It is generally thought that vertebrate ROBO1 corresponds closest in function to this ancestral gene.

### The two genomic clusters of vertebrate roundabouts

What is the genomic arrangement of the four human genes encoding roundabouts? ROBO4 is tightly clustered (in a window of 40 kilobases) with ROBO3 in a tail-to-tail arrangement located on chromosome 11 (S1 Fig, Table 1). Interestingly, ROBO1 and ROBO2 are also clustered, in an analogous tail-to-tail arrangement, but located on chromosome 3 (and this cluster is not as compact). See S1 File, section *The conservation of roundabout clusters*, for an estimate the frequency of the conservation of a two-gene human cluster similar to ROBO3-ROBO4 throughput vertebrates.

**Table 1:** The clustered arrangement of roundabouts is conserved in vertebrates.

Species	The genomic clusters of roundabouts*					
	ROBO3-ROBO4			ROBO2-ROBO1		
	Size	Chr.	Boundaries	Size	Chr.	Boundaries
*Homo sapiens*	30 kb	11	124.87–124.9 Mb;	3.87 Mb	3	75.9–79.77 Mb;
*Mus musculus*	30 kb	9	37.4–37.43 Mb;	1.75 Mb	16	72.65–74.4 Mb;
*Felis catus*	35 kb	D1	21.72–21.75 Mb;	1.8 Mb	C2	30.69–32.5 Mb;
*C*. *lupus familiaris*	32 kb	5	9.55–9.58 Mb;	1.5 Mb	31	8.1–9.6 Mb;
*Bos taurus*	46 kb	29	28.69–28.74 kb;	1.65Mb	1	24.35–26 Mb
*Equus caballus*	26 kb	7	33.4–33.43 Mb;	1.4 Mb	26	10.5–11.9 Mb;
*X*. *tropicalis*	185 kb	scaffold GL172933	1.015–1.2 Mb;	0.6 Mb	scaffold GL172645	0.75–1.35 Mb;
*Danio rerio*	10 kb	10	3.135–3.15 Mb;	0.8 Mb	15	38.95–39.75 Mb**.

* In all the species, the arrangements of genes within both clusters are tail-to-tail

** In a tail-to-tail arrangement, but there is an additional copy of robo2 downstream of the cluster.

NOTE: Genomic locations are given in the coordinates of the hg38 assembly.

### The *tandem-plus-WGD* model of the emergence of vertebrate roundabout clusters

The TreeBest phylogenetic tree (Fig 2) must be interpreted through the lens of the clustered arrangement of vertebrate roundabouts. Why are ROBO3 and ROBO4 grouped in a tight genomic cluster if both these sequences are early diverging rather than a pair of tandem duplicates? (This robust topology could be observed not only for the heuristic TreeBeST tree, but also for Bayesian trees calculated using multiple models of rate variation between sites, and separately for both the intracellular and extracellular regions of roundabouts.) A two-step model with accelerated evolution of ROBO3 and ROBO4 reconciles the three lines of somewhat conflicting evidence: the tree topology, phylogenetic timing, and genomic clustering. In this model, a tandem duplication was the first step. The second step was the duplication of the tandem cluster through 2R-WGD. We note that there are only two (rather than four) roundabout clusters in vertebrates. The two other copies derived from 2R-WGD must have been lost during rediploidization. We label the above two-step model: *tandem-plus-WGD*. Several elements of empirical evidence support *tandem-plus-WGD*:

1. the fact that all three duplication nodes of vertebrate roundabouts, Fig 2, are dated by phylogenetic timing to the base of vertebrates when 2R-WGD occurred and when ECs emerged as a novel cell type;
2. the tail-to-tail arrangement of both genomic clusters of vertebrate roundabouts indicating they might be the duplicates of each other;
3. the fact that 2R-WGD ohnologs are preferentially involved in signal transduction.

In the above model, the topology of the tree reflects not the temporal order of duplications, but the accelerated evolution and asymmetric divergence of ROBO4.

A model similar to *tandem-plus-WGD* was suggested for ROBO 1/2/3 by other authors [35]. Zelina *et al*. differentiate between two alternative sub-models, depending on whether the losses of clusters occurred after the first (R1) or the second (R2) round of 2R-WGD. We note that the loss after R1 is more parsimonious assuming just a single deletion. (The loss after R2 would require two deletions.) Zelina *et al*. did not comment on the topology of the ROBO4 branch as ROBO4 was not included in their phylogenetic tree. However, Zelina *et al*. did detect positive selection in the mammalian [sic ] sequences of ROBO3. The authors proposed that the function of ROBO3 shifted from a receptor for SLIT repulsion into a receptor that silences SLIT repulsion and enhances attraction to netrin. The mammalian-specific substitutions were implicated in ROBO3’s lost the ability to bind SLIT. We note that the observation of positively selected sites in ROBO3 is in agreement with the increased length of its branch in Fig 2 (in relation to the branches of ROBO1 and ROBO2).

The early diverging topology of the ROBO4 branch in Fig 2 could also be an artifact called long branch attraction—LBA. Fast evolving and asymmetrically diverging paralogs can be grouped as sister groups with more diverged branches because of LBA, which occurs especially if a tree is a mixture of orthologs and paralogs [36, 37]. It was demonstrated specifically for yeast WGD that LBA may cause incongruent topologies among ohnologs [38]. We note that the early-diverging topology of the ROBO4 branch persisted in a MrBayes tree using the gamma distribution of rates which was suggested by Fares *et al*. to ameliorate LBA in some cases.

For the discussion of an alternative model see section entitled *An alternative model* in S1 File.

### The functional protein interactions of ROBO4

**Fig 3:**
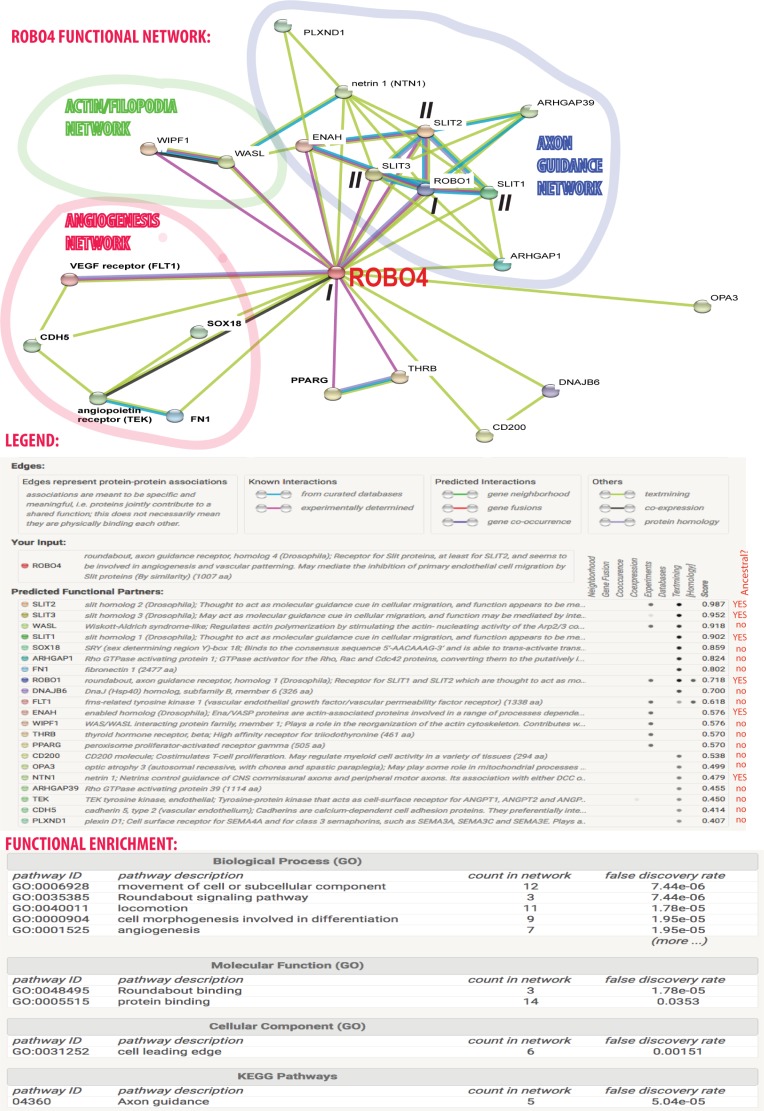
ROBO4 integrates several functional networks including a neo-functionalized network regulating angiogenesis. ROBO4 is an endothelial-specific network hub, and a signaling bridge which integrates three functional sub-networks of the vertebrate cell: (1) the angiogenesis network, (2) the actin/filopodia network, and (3) the axon guidance network. The legend beneath the network graph provides information on sources of evidence and scores obtained for the interactors. Functional enrichment analysis at the bottom of the figure provides information on enriched gene ontology terms and KEGG pathways. Roman numbers indicate two sets of ohnologs: ROBO1 and ROBO4 (I) and SLIT1-3 (II).

In the following sections, various aspects of the divergence of roundabout ohnologs are investigated. We start with protein interactions for which the role of gene duplications, especially the resolution of the network after WGDs, are still incompletely understood. To investigate ROBO4 from a network perspective, the STRING database of functional protein interactions was queried (Fig 3). The database integrates several types of experimental evidence and computational predictions [39–41]. Many of the interactions of ROBO4 that were published in molecular biology literature (reviewed in Table 2) can be found in the network shown in Fig 3. For example, the interactions of ROBO4 with ROBO1 and with FLT1 are present. These interactions have the support of multiple lines of evidence in the STRING database (from text-mining, high-throughput experiments, and homology). While such experimentally-determined interactions of ROBO4 could be found in original publications, the STRING database facilitates meta-analysis and network interpretation that would not be possible otherwise.

**Table 2:** Experimentally-verified protein interactions of ROBO4 in human and mouse.

Interactor	Interactant	Assay	Reference (sorted by the year of publication)
Human **ROBO4**	Slit guidance ligand 2 (**hSLIT2**), enabled homolog (**hENAH**);	Co-immunoprecipitation;	[72];
Human **ROBO4**	Retinoid X receptor alpha (**hRXRA**), peroxisome proliferator activated receptor gamma (**hPPARG**), thyroid hormone receptor beta (**hTHRB**);	*In vitro* pull-down;	[103];
Mouse **Robo4**	Slit guidance ligand 3 (**mSlit3**);	Co-immunoprecipitation;	[104];
Mouse **Robo4**	Paxillin (**PXN**);	Yeast two hybrid;	[105];
Human **ROBO4**	Roundabout 1 (**hROBO1**), Wiskott-Aldrich syndrome protein (**hWASP**), neural Wiskott-Aldrich syndrome protein (**hWASL**), WAS/WASL-interacting protein family member 1 (**hWIPF1**);	Yeast two hybrid, *in vitro* pull-down;	[106];
Mouse **Robo4**	Unc-5 Netrin Receptor B (**hUnc5b**);	Surface plasmon resonance, co-immunoprecipitation;	[75];
Human **ROBO4**	Fms related tyrosine kinase 1 (**hFLT1, VEGFR1**);	Co-immunoprecipitation;	[107].

Interestingly, the interaction network suggests that ROBO4 is a network hub (with 21 edges) and a bridge [42] integrating three functional sub-networks. (Such bottlenecks are attractive targets for pharmacological intervention, as there is little signaling robustness. In other words, if a bottleneck-type node is knocked-out, there are no alternative paths of the flow of information.) The first sub-network controls angiogenesis. This network includes: the VEGF receptor FLT1, vascular endothelial cadherin (CDH5), the TEK angiopoietin receptor, fibronectin 1 (FN1), and the TF SOX18. The second sub-network controls the formation of filopodia: Wiskott-Aldrich syndrome-like (WASL) and WAS/WASL interacting protein family member 1 (WIPF1). The third sub-network controls axon guidance: roundabout 1 (ROBO1), three Slit genes (SLIT1-3), netrin 1, two Rho GTPase activating proteins (ARHGAP1 and ARHGAP39), enabled homolog (ENAH), and a cell surface receptor for class 3 semaphorins named plexin D1 (PLXND1). This is congruent with molecular and cell biology literature which suggests that actin-supported filopodia are created by both the growth cones of neuronal axons [43] and by migrating angiogenic endothelial tip cells [44].

Unlike ROBO4, ROBO1 is not a network bridge. Instead, it is embedded entirely within the axon guidance sub-network with most of interactors corresponding to the ancestral network of robo1 in the *D*. *melanogaster*. However, ROBO1 and ROBO4 share some interaction partners (namely ENAH, netrin 1, SLIT1-3, ARHGAP1 and ARHGAP39) which is common for paralogs. Nevertheless, the majority, 12, of ROBO4’s interaction partners are not shared with ROBO1.

The key test of the neo-functionalization model for network interactions is the comparison of the networks of paralogs with the ancestral network. To estimate the ancestral pre-2R-WGD interaction network, we searched the STRING database for the interactions of robo1 in *D*. *melanogaster*. Ten interacting proteins were found: slit (sli), Abl tyrosine kinase (Abl), leak (lea), enabled (ena), netrin A and B (NetA and NetB), dally-like (dlp), robo3, commissureless (comm), and Eph receptor tyrosine kinase (Eph). Note that robo3 is a fly-specific paralog of robo, it is not a 1-to-1 ortholog of vertebrate ROBO3. Because of the fly-specific paralogs, the estimate of the ancestral network is not optimal but it is the best possible with the data available today. An alternative would be to use the network of *C*. *elegans*, where there is only one roundabout ortholog: sax3, but because of the worm’s unique development it’s signaling pathways are typically rather diverged.

The majority ROBO4’s interactions do not correspond to the ancestral network of robo1, suggesting they were acquired after 2R-WGD, during ROBO4’s asymmetric divergence. Similar conclusions are reached if experimental interactions of ROBO4 (Table 2) are examined instead of the STRING network. In Table 2, only SLITs, the enabled homolog, and ROBO1 correspond to the ancestral network. The other interactions are neo-functionalizations. In contrast, most ROBO1’s edges can be interpreted as orthologs of robo1’s edges, if one takes into account that vertebrate SLIT1-3 are ohnologs with the fly’s sli as the out-group.

### The expression patterns of roundabouts in F5

F5 is not only more comprehensive than functional genomics resources available before, but it also allows the discovery of alternative transcription start sites (TSSes) at single-base resolution which has never been performed for roundabouts. Comprehensiveness is crucial when investigating tissue-specificity; if only a few cell- or tissue-types are examined, alternative sites of expression may be missed leading to false presumption of tissue-specific expression.

**Fig 4:**
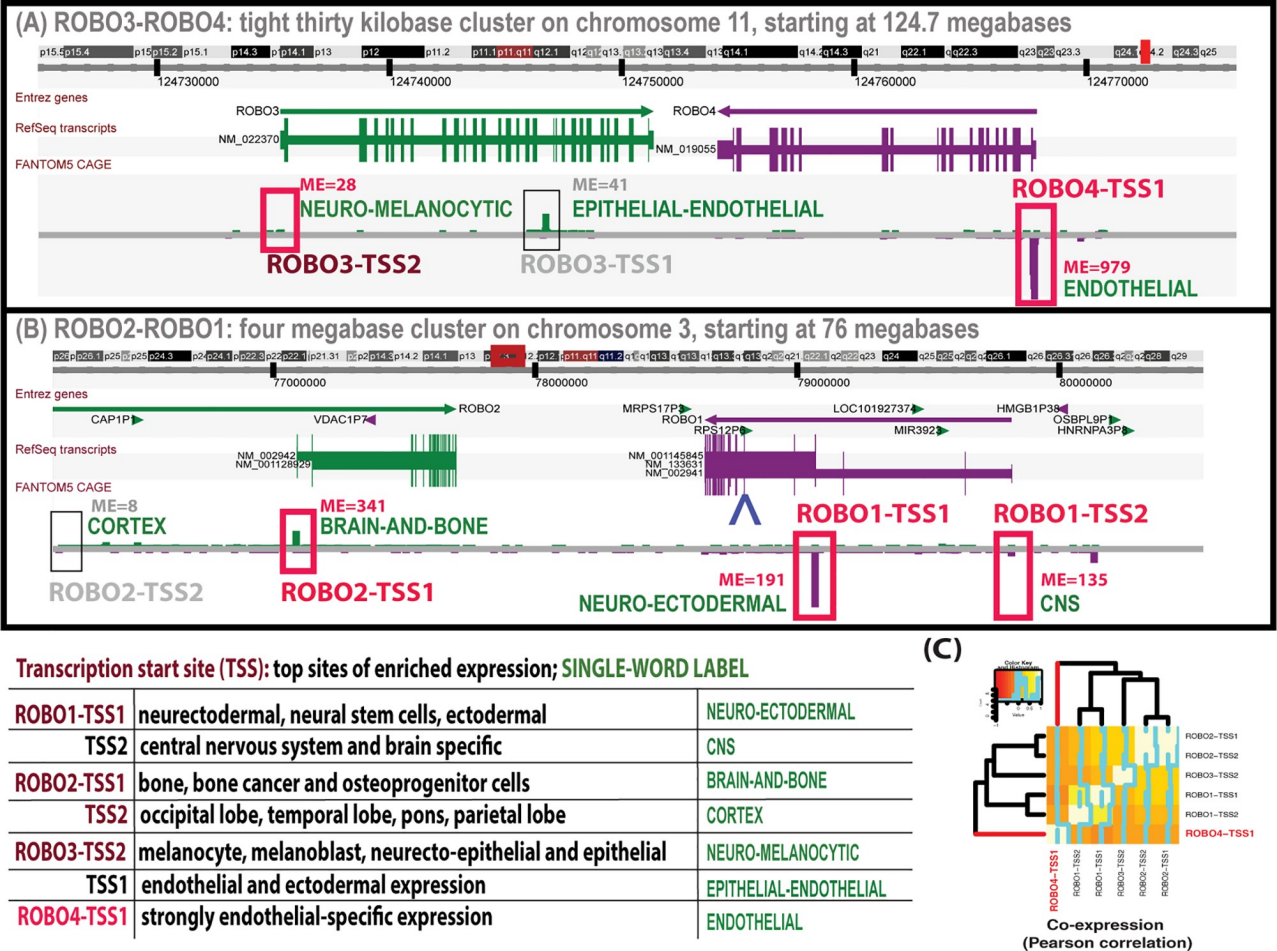
The expression profiles of the roundabout TSSes. Alternative TSSes were identified in the ROBO3-ROBO4 (A) and ROBO2-ROBO1 (B) clusters based on location with reference to the beginnings of RefSeq transcripts and maximal expression (ME), in that order. Expression profiles were inferred from human F5 data and TSSes visualized using the Zenbu browser. For example, ROBO3 has two alternative TSSes: one is expressed in melanocytes and epithelial cells (ROBO3-TSS2). The other is characterized by endothelial and neuroectodermal expression (ROBO3-TSS1). In contrast, ROBO4 has one (ROBO4-TSS1) sharply defined strong endothelial-specific TSS. (Graphical gene models of roundabouts derive from the UCSC Genome Browser, modified with Adobe Illustrator). The boxes of TSSes which are conserved in mouse are highlighted in red. Note that the ROBO4 transcript is 5’-truncated in relation to the other roundabouts. Protein homology to the ROBO4 protein starts only at the third exon of the transcript of ROBO1-TSS1 (ENSEMBL exon ID ENSE00001149757, marked with the blue `^`sign by the gene models of ROBO1). In panel (C), a heatmap of pairwise Pearson correlation coefficients visualizes the co-expression between roundabout TSSes in F5 primary cells. ROBO4-TSS (highlighted in red) is dissimilar in expression and clusters as an out-group to the expression profiles of other TSSes.

The results of the analysis of F5 suggest that ROBO4 has only one TSS which is strongly expressed in endothelial cells (Fig 4). How strongly? As shown in Table 3, close to one in a thousand expression tags is derived from the single TSS of ROBO4 (labeled here ROBO4-TSS1) in renal glomerular ECs. This endothelial expression was not only strong but also very specific to the endothelial cell type. The RefSeq linked with ROBO4-TSS1, namely NM_019055, was the 16^th^ most enriched transcript in the group of F5 endothelial libraries (ranked in the top 99.9 percentile of enrichment; see methods for details on the endothelial libraries). The transcript was 98-fold enriched in the endothelial libraries (with the average expression of 1188 TPM). We note that it is known that Robo4 also has some expression in non-endothelial cells, for example in hematopoietic cells [45]. However, this is not surprising given that endothelial cells share common origins with blood cells, both in terms of the evolution [46, 47] and in term of the development [48, 49]. (We note that endothelial-specificity of ROBO4-TSS1 is conserved in mouse where the top site of expression is in liver sinusoidal ECs—304 TPM. The second and third most abundant sites were hepatic stellate cells and neonate lung with the expression signals of only 57 and 42 TPM, respectively.)

**Table 3:** The expression profiles of the TSSes of roundabouts. For each TSS, we show either the expression signal in each individual library (bold font) or enrichment in the sets of human F5 samples grouped in sample ontologies.

GENE-TSS (SINGLE-WORD LABEL)	The location of the TSS (1kb interval, strand)	The top five tissues of expression with signal in in tags per million (TPM); or z-score of enrichment in a sample category* (with the number of samples in the category given in curly brackets)		
ROBO4-TSS1 (**ENDOTHELIAL**)	chromosome 11: 124767260–124768260 (-)	**TPM**	**renal glomerular endothelial cells 1**	**979**
			**endothelial cells–microvascular**	**662**
			**endothelial cells–aortic**	**577**
			**endothelial cells–microvascular**	**563**
			**renal glomerular endothelial cells 2**	**544**
		*z-score*	*endothelial cell of vascular tree*, *CL*:*0002139 (69)*	*14*
			*endothelial cell*, *CL*:*0000115 (83)*	*14*
			*tissue sample*, *FF_ont*:*0000004 (198)*	*14*
			*meso-epithelial cell*, *CL*:*0002078 (93)*	*14*
			*endothelial cell of lymphatic vessel*, *CL*:*0002138 (51)*	*13*
ROBO3-TSS2 (**NEURO-MELANOCYTIC**)	chromosome 11: 124734800–124735800 (+)	**TPM**	**H9 embryoid body cells, melanocytic induction, day12, oil rep1, tech rep1**	**28**
			**H9 embryoid body cells, melanocytic induction, day12, oil rep1, tech rep2**	**28**
			**H9 embryoid body cells, melanocytic induction, day27, oil rep1**	**14**
			**H9 embryoid body cells, melanocytic induction, day09, oil rep1**	**12**
			**merkel cell carcinoma cell line**	**10**
		*z-score*	*melanocytic induction*, *FF_ont*:*0000334 (44)*	*7*
			*H9 embryonic stem cell line*, *FF_ont*:*0000400 (47)*	*7*
			*melanoblast*, *CL*:*0000541 (53)*	*6*
			*neurecto-epithelial cell*, *CL*:*0000710 (64)*	*6*
			*neurectodermal cell*, *CL*:*0000133 (155)*	*6*
ROBO3-TSS1 (**EPITHELIAL-ENDOTHELIAL**)	chromosome 11: 124746200–124747200 (+)	**TPM**	**endometrial stromal sarcoma cell line**	**41**
			**nasal epithelial cells**	**35**
			**small airway epithelial cells**	**35**
			**fibroblast–conjunctival**	**35**
			**epithelioid sarcoma cell line**	**34**
		*z-score*	*cell by lineage*, *CL*:*0000220 (365)*	*12*
			*embryonic cell*, *CL*:*0002321 (457)*	*11*
			*endothelial cell of vascular tree*, *CL*:*0002139 (69)*	*10*
			*ectodermal cell*, *CL*:*0000221 (170)*	*9*
			*endothelial cell of lymphatic vessel*, *CL*:*0002138 (51)*	*9*
ROBO2-TSS2 (**CORTEX**)	chromosome 3: 75955300–75956300 (+)	**TPM**	**occipital lobe**	**8**
			**temporal lobe**	**8**
			**pons, adult**	**8**
			**parietal lobe**	**8**
			**mucinous adenocarcinoma cell line**	**0**
		*z-score*	*parietal lobe–adult*, *FF_ont*:*0010034 (3)*	*2*
			*medulla oblongata–adult*, *FF_ont*:*0010038 (3)*	*2*
			*locus coeruleus- adult*, *FF_ont*:*0011487 (3)*	*2*
			*fibroblast of periodontium*, *CL*:*0002556 (6)*	*1*
			*motile cell*, *CL*:*0000219 (756)*	*-1*
ROBO2-TSS1 (**BRAIN-AND-BONE**)	chromosome 3: 77088794–77089794 (+)	**TPM**	**pineal gland, adult**	**341**
			**cerebellum, newborn**	**140**
			**parietal lobe, fetal**	**132**
			**gastric cancer cell line:AZ521**	**131**
			**temporal lobe, fetal**	**126**
		*z-score*	*SAOS-2 cell (FF_ont*:*0100971) (57)*	*11*
			*calcification induced with ascorbate and BPG (FF_ont*:*0000332) (54)*	*10*
			*osteosarcoma cell line (FF_ont*:*0100407) (59)*	*10*
			*bone cancer cell line (FF_ont*:*0100385) (61)*	*10*
			*osteoprogenitor cell (CL*:*0000375) (64)*	*9*
ROBO1-TSS1 (**NEURO-ECTODERMAL**)	chromosome 3: 79068100–79069100 (-)	**TPM**	**gastrointestinal carcinoma cell line**	**191**
			**neural stem cells**	**108**
			**COBL-a rinderpest(-C) infection, 48hr**	**78**
			**occipital lobe, fetal**	**90**
			**xeroderma pigentosum b cell line**	**80**
		*z-score*	*neurectodermal cell*, *CL*:*0000133 (155)*	*11*
			*ectodermal cell*, *CL*:*0000221 (170)*	*11*
			*non-terminally differentiated cell*, *CL*:*0000055 (402)*	*10*
			*pigment cell*, *CL*:*0000147 (82)*	*9*
			*H9 embryonic stem cell line*, *FF_ont*:*0000400 (47)*	*9*
ROBO1-TSS2 (**CNS**)	chromosome 3: 79816700–79817700 (-)	**TPM**	**temporal lobe, fetal, donor1, tech rep2**	**135**
			**temporal lobe, fetal, donor1, tech rep1**	**94**
			**occipital lobe, fetal, donor1**	**64**
			**non-small cell lung cancer cell line**	**62**
			**nucleus pulposus cell, donor1**	**48**
		*z-score*	*locus coeruleus- adult*, *FF_ont*:*0011487 (3)*	*3*
			*spinal cord–adult*, *FF_ont*:*0010159 (3)*	*3*
			*fetal tissue sample*, *FF_ont*:*0000999 (28)*	*3*
			*H9 embryoid body cells 12 days after melanocytic induction*, *FF_ont*:*0000537 (5)*	*3*
			*melanocytic induction*, *FF_ont*:*0000334 (44)*	*2*

* Groupings of samples derive from the following sample ontologies: UBERON, or Gene Ontology. Ontologies are sorted according to the Wilcoxon-mann-whitney rank-sum enrichment *z*-score. The top five enriched sample categories are given for each TSS.

Note: locations are given in the coordinates of the hg19 assembly.

In contrast, all the other roundabout-linked RefSeqs were depleted in endothelial cells (that is the average expression in the endothelial libraries was lower than in non-endothelial ones). However, the ROBO1-TSS2-linked transcript was still fairly abundant in the endothelial libraries (the average of 93 TPM). The transcript of ROBO1-TSS1 had the average endothelial expression of only 10 TPM. The other roundabout transcripts were absent from ECs (their endothelial expression was lower than 1 TPM). We note that the endothelial expression of ROBO1 could also be interpreted as partial neo-functionalization, although this ohnolog conserved the ancestral neuronal expression site. A general increase in the rate of expression divergence of orthologs in the presence of paralogs was reported previously [11].

The expression pattern driven by ROBO-TSS1 clearly stands out. As expected under the neo-functionalization model, it is not correlated with those driven by the other human roundabout TSSes (Fig 4*C*, Table 4). In fact, if a *P*-value is calculated using a randomization test appropriate for estimating the genomic background / random expectation for a co-expression analysis (see methods), ROBO-TSS1’s expression pattern is actually significantly anti-correlated with those of ROBO3-TSS1/2, ROBO2-TSS1/2 and ROBO1-TSS1. These other TSSes, however, do correlate with each other and their top sites of expression are mostly in the central nervous system—CNS (can be presumed to be neuronal). Note that the expression of the *Drosophila* roundabout, robo1, is also neuronal [50] proving this was the ancestral state in the last common ancestor of vertebrates and insects. Clearly, it was the expression pattern of ROBO4 which shifted in relation to the ancestral state. In other words, ROBO4 neo-functionalized on the level of expression pattern.

**Table 4:** ROBO4-TSS1 does not correlate in expression with the other roundabout TSSes. Pearson correlation coefficients (PCCes) for expression profiles in human F5 libraries from primary cells are given. Both asymptotic P-values (PA) and P-values from sampled randomization (PR) are shown. Interestingly, the negative PCCes of ROBO4 are not significant in the asymptotic test, but are significant in the randomization test*.

SS	RefSeq	ROBO3-TSS1/2	ROBO2-TSS2	ROBO2-TSS1	ROBO1-TSS1	ROBO1-TSS2
ROBO4-TSS1	NM_019055	**PCC = -0.03,** **P**_**A**_ **= 0.48,** **P**_**R**_ **= 0.009**	**PCC = -0.03** **P**_**A**_ **= 0.57,** **P**_**R**_ **= 0.009**	**PCC = -0.04** **P**_**A**_ **= 0.43,** **P**_**R**_ **= 0.001**	**PCC = -0.08** **P**_**A**_ **= 0.09,** **P**_**R**_ **= 0**	PCC = -0.01, P_A_ = 0.749, P_R_ = 0.234
ROBO3-TSS1/2	NM_022370		***PCC = 0*.*22*,** ***P***_***A***_ ***= 1e-06*,** ***P***_***R***_ ***= 0*.*012***	***PCC = 0*.*22*,** ***P***_***A***_ ***= 1e-06*,** ***P***_***R***_ ***= 0*.*012***	***PCC = 0*.*13*,** ***P***_***A***_ ***= 0*.*0044*,** ***P***_***R***_ ***= 0*.*033***	PCC = 0.03, P_A_ = 0.43, P_R_ = 0.209
ROBO2-TSS2	NM_001128929			***PCC = 0*.*87*,** ***P***_***A***_ ***< 2*.*2e-16*,** ***P***_***R***_ ***= 0***	***PCC = 0*.*25*,** ***P***_***A***_ ***= 1*.*271e-08*,** ***P***_***R***_ ***= 0*.*009***	***PCC = 0*.*18*,** ***P***_***A***_ ***= 4*.*352e-05*,** ***P***_***R***_ ***= 0*.*018***
ROBO2-TSS1	NM_002942				***PCC = 0*.*27*,** ***P***_***A***_ ***= 6*.*991e-10*,** ***P***_***R***_ ***= 0*.*008***	***PCC = 0*.*18*,** ***P***_***A***_ ***= 2*.*869e-05*,** ***P***_***R***_ ***= 0*.*018***
ROBO1-TSS1	NM_133631					***PCC = 0*.*72*,** ***P***_***A***_ ***< 2*.*2e-16*,** ***P***_***R***_ ***= 0***

NOTE: PCCes significant in the randomization test are highlighted in bold. Those which are significant in both the randomization test and the asymptotic test are highlighted in both bold and italics.

* The randomization test rejects a data-conditioned null hypothesis: that the value of correlation is not different from the distribution obtained for the genomic background.

Moreover, roundabouts differ more than hundred-fold in their maximal expression (ME), that is the highest expression level attained in any tissue- or cell-type. ROBO4-TSS1 has a very potent ME of 979 TPM (Table 3). The values for other roundabout TSSes are lower, varying from 341 to 28. ME for ROBO2-TSS2 is on the verge of illegitimate transcription (8 TPM). ME is very informative as it gives a measure of the maximal transcriptional output of a promoter, in other words it answers the question of how strong given promoter can potentially be. The above values again underlined how different was expression pattern of ROBO4 was in comparison to other roundabouts.

### Can the neo-functionalized expression pattern of ROBO4 be explained by the architecture of the proximal promoter of ROBO4?

As ROBO4 neo-functionalized to endothelial-specific expression, we compared the architecture of its proximal promoter with those of other roundabouts in search for clues of how this expression shift was effected. Using ENCODE data for human umbilical vein endothelial cells—HUVECs (S2*A* Fig, Table 5), we found the following strong endothelial TFBSes in the three-kilobase window upstream of ROBO4-TSS1: CCCTC-binding factor (CTCF), Fos Proto-Oncogene (FOS), Jun proto-oncogene (JUN) and GATA binding protein 2 (GATA2). Additionally, there was a weak endothelial TFBS for MYC (QS = 119) and a number of overlapping non-endothelial TFBSes, most of which were weak sites, grouped together in two clusters (proximal and distal to ROBO4-TSS1). The strong GATA2 site (with quality score—QS equaling 704) overlapped with a DNA region highly conserved in vertebrates, and is likely to be a key controller of endothelial-expression of ROBO4. MYC is now thought of as an amplifier of expression patterns already established in the cell [51, 52]. Although ROBO1-TSS1 also had a GATA2 site, it was a weak non-EC site (QS = 301) from a cancer cell-line.

**Table 5:** Promoter architectures associated with TSSes of roundabouts. Strong sites* are shown in **bold font**. Those detected in ECs are marked with (+). GATA2 and GATA3, as well as AP-1 subunits (FOS and JUN-family) TFBSes are underlined. The sites do not include polymerase type II peaks whose number correlates trivially with the level of gene expression. We note that in some loci high-affinity sites were shown to be inhibitory while low-affinity sites were shown to activate transcription [108]. This is not the case for ROBO4 as most of its strong TFBSes were activating endothelial sites. However, several weak sites of ROBO4-TSS1 were also of endothelial origin, suggesting both strong and weak TFBSes could be functional in this promoter architecture.

ENCODE TFBSes.	TSS.
***CTCF (+)*,** ***FOS (+)***, ***GATA2 (+)***, ***JUN (+)*****, CEBPB, REST, STAT3;** *CTCF (+)*, *GATA2 (+)*, *JUN (+)*, *MYC (+)*, EP300, FOSL2, FOXA1, FOXP2, JUND, MAFK, REST, SIN3AK20, SP1, STAT3, TCF7L2;	ROBO4-TSS1
***CTCF (+)*, *EZH2 (+)*, E2F6, MAX, RAD21, REST, USF1;** *CTCF (+)*, CTCFL, EGR1, ESR1, EZH2, FOXA1, MYC, REST, SETDB1, SMC3, SP2, SUZ12, USF2, YY1, ZBTB7A, ZNF143;	ROBO3-TSS2
***CTCF (+)*, EGR1, GABPA, RAD21, ZNF143;** BACH1, CBX3, CCNT2, CREB1, CTBP2, CTCFL, E2F6, ELF1, ELK1, ELK4, EP300, ETS1, FOXP2, HMGN3, JUND, MAX, MAZ, MXI1, MYC, PAX5, PHF8, RAD21, RBBP5, REST, SIN3A, SIN3AK20, SMC3, SP2, SP4, TAF1, TAF7, TBP, THAP1, UBTF, YY1, ZBTB7A;	ROBO3-TSS1
**CTCF(+), RAD21, TCF7L2;** CEBPB, E2F6, EGR1, EP300, EZH2, FOXP2, HDAC2, MAX, MXI1, RAD21, RBBP5, SMC3, STAT1, TAF1, TBP, USF1	ROBO2-TSS1
**CTCF(+), RAD21, ZNF263;** EGR1, RBBP5, TAF1, TBP, TEAD4, YY1, ZNF143	ROBO2-TSS2
**CTCF,** **JUND****, BHLHE40, CEBPB, CHD1, EP300, MAFK, TAF1, TBP;** *CTCF (+)*, ARID3A, ATF2, BACH1, BRCA1, CEBPD, CTBP2, E2F4, EBF1, EGR1, ELF1, EP300, FOS, FOXA1, FOXP2, GATA2, GATA3, GTF2F1, HDAC2, KAP1, MAFF, MAX, MAZ, MXI1, MYBL2, MYC, NFIC, RAD21, RBBP5, RCOR1, RELA, REST, RFX5, SIN3A, SIN3AK20, SIX5, SMARCC2, SMC3, SP1, SPI1, SRF, STAT1, STAT3, TAF1, TCF12, TCF7L2, TEAD4, TFAP2C, USF1, ZBTB33, ZNF263	ROBO1-TSS1
**CTCF;** *CTCF (+)*, BACH1, ESR1, EZH2, FOSL1, HDAC2, SP1, USF1, ZNF143	ROBO1-TSS2

* The quality score cutoff of 500 divided ENCODE TFBSes into strong and weak sites.

None of the other roundabout promoter had either a similar set of TFBSes or the similar structural arrangement into proximal and distal clusters as ROBO4-TSS1 promoter did. In the S2*B* and S2*C* Fig, two additional promoter architectures, of which ROBO4-TSS1 could potentially be a duplicate, were visualized: ROBO3-TSS2 and ROBO1-TSS1. (The other roundabout promoters are shown in S3 Fig). It is striking that there is no similarity of ROBO4-TSS1 to any of these promoters: neither in the set of TFs they can bind, nor in the arrangement of conserved regions, nor in the type of canonical DNA binding motifs. The architecture of ROBO4-TSS1 was also the only one with a high proportion of endothelial TFBSes (four out of 7 strong sites). All these observations are agreeable with the model of ROBO4’s neo-functionalized endothelial-specific expression to be controlled by a new promoter architecture which has no similarity to the ancestral one. We note that the promoters are also not similar on nucleotide level. BLAT [53] searches against the genome revealed only self-hits in the regions of both roundabout clusters. However, this was expected as vertebrate duplications are too old for a conservation of non-coding DNA sequences. Arguably, nucleotide-level comparisons of such promoters are incorrect methodologically.

### Accelerated evolution in the ROBO4 lineage and amino-acid sites under positive selection

ROBO4 was included among genes under positive selection in the dolphin genome [54]. This observation and the detection of positively selected sites in ROBO3 [35], prompted us to examine the rate of evolution on the ROBO4 branch. Two strategies implemented in the PAML 4 package were employed. Firstly, the rates of non-synonymous (*k*_*A*_) and synonymous (*k*_*S*_) substitutions were calculated on different branches the roundabout tree and their ratio was used to estimate the average evolutionary rate for the entire sequence length. However, the rate of evolution over the entire length of the coding sequence is very rarely higher than one, even if the protein is positively selected. Therefore, individual sites of positive selection among the codons of ROBO4 were searched for using variable ratio [55] and branch-site models implemented in PAML [56, 57]. The results are displayed in Table 6. Furthermore, PAML uses the framework of maximum likelihood allowing individual models to be compared using likelihood ratio tests—LRTs. The results of these tests are displayed in Table 7. The conclusions are that ROBO4 evolved at an accelerated rate and that many of its codons were under positive selection after 2R-WGD and before the human-mouse split. We note that positive selection is expected under the neo-functionalization model as it assumes that the evolution of a paralog is driven by adaptation.

**Table 6:** Accelerated evolution and positively selected sites in ROBO4. The table shows the values of log likelihoods (l) and the estimates of parameters under different models of the rate of evolution among the codons of ROBO4. The models were applied to the small dataset only. The first group of models average the rates of evolution over the entire tree. The model assuming one parameter (*i*.*e*., the one-ratio model) calculates $\omega =\frac{d_{N}}{d_{S}}$, which equaled 0.1095, for the entire tree. This model was least likely (l=−14869). The next group of models allow the rate of evolution to vary between branches. The free-ratio model calculates a separate rate of evolution for each branch. The branch model calculates one rate of evolution for the ROBO4 branch (foreground) and the other for the remaining branches (background). The branch-site model allows the rate of evolution to vary both among sites and between the foreground and background.

Model		*p*	l	The estimates of parameters	Positively selected sites
**Rates do not vary between branches**	**One-ratio**	1	-14869	${\hat{\omega }}_{0}=0.11$	Not allowed
	**Nearly-neutral**	2	-14849	${\hat{\omega }}_{0}=0.1\;(\omega =1)$ *p*_0_ = 0.94, *p*_1_ = 0.06	Not allowed
	**Positive-selection**	4	-14849	${\hat{\omega }}_{0}=0.1\;(\omega =1),$ ${\hat{\omega }}_{2}=40.1,p_{0}=0.94$ *p*_1_ = 0.059,*p*_1_ = 0	None
	**Beta**	2	-14714	*p* = 1.08,*q* = 7.9	Not allowed
	**Beta & *ω* > 1**	5	-14715	*p*_0_ = 1,*p* = 1.08 *q* = 7.9 (*p*_1_ = 0) ${\hat{\omega }}_{S}=6.44$	None
**Free-ratio model** (one *ω* for each branch)		15	-14790	${\hat{\omega }}_{ROBO4}=0.59$	Not allowed
**Branch model** (the ROBO4 branch as foreground)		2	-14864	${\hat{\omega }}_{0}=0.11,$ ${\hat{\omega }}_{1}=0.54$	Not allowed
**The branch-site model A (null)**		2	-14830	${\hat{\omega }}_{0}=0.1\;(\omega =1)$ *p*_0_ = 0.49, *p*_1_ = 0.03	Not allowed
**The branch-site model A**		4	-14808	${\hat{\omega }}_{0}=0.1\;(\omega =1)$ ${\hat{\omega }}_{2}=6.9,p_{0}=0.42$ *p*_1_ = 0.02	58 sites (BEB>95%). 12 sites (BEB>99%) out of 630

NOTE: *p* stands for the number of parameters under different models. l stands for log-likelihood. Branch lengths are fixed at their maximum likelihood estimates under the one-ratio model. The sites of positive selection are inferred at the BEB score higher than 95%.

**Table 7:** Likelihood ratio tests on PAML data.

Models compared	2Δl	D.f.	Chi -squared	Conclusions
One-ratio *vs*. free-ratio	79	14	*P* = 2e-11	Rejects the one-ratio model
One-ratio *vs*. branch model	10	1	*P* = 0.00085	Rejects the one-ratio model
Branch-site null *vs*. branch-site	44	2	*P* = 0	Rejects the branch-site null model

## Discussion

The analyses performed herein prove that ROBO4 neo-functionalized on the level of gene expression. Considered as a group, vertebrate ROBO1/2 and 3 should be labeled *neuronal*. ROBO4, in contrast, is powerfully and specifically expressed in ECs from a single TSS (ROBO4-TSS1) identified here using F5. Drosophila’s *robo*, the single invertebrate roundabout ortholog [50], represents the ancestral function in axon guidance. It also represents the ancestral mode of expression in neuronal tissue. However, unlike many known mammalian examples of expression neo-functionalization [58, 59], ROBO4 is not an intronless passenger of retrogene activation in testis. Neither is it the result of gene traffic between chromosome X and autosomes enforced by the cap on ME on chromosome X due to haploidy [60]. Instead, ROBO4’s shift in expression pattern was a functional adaptation driven by evolutionary emergence of vascular endothelium.

What is exceptionally interesting about ROBO4 is that its emergence was entangled with the emergence of the novel cell type: the endothelial cell. As there is no true endothelium in invertebrates [5], this cell type could not have been a part of the ancestral roundabout expression pattern. Even amphioxus, despite its close relationship to vertebrates, has no ECs [47]. Along with the EC cell type came a novel set of regulatory pathways controlling angiogenesis and vascular integrity / permeability, including the interaction network of ROBO4. As the evolutionary origins of the endothelium and these signaling pathways are incompletely understood, molecular evolutionary evidence is of much value.

If ROBO4’s expression pattern is a neo-functionalization, how is it regulated? Okada *et al*. proved the importance of the three kilobase (upstream of the TSS) proximal promoter of ROBO4 for controlling its endothelial-specific expression [61]. Okada *et al*. used deletions, electrophoretic mobility shift assays and chromatin immunoprecipitation assays to analyze the proximal promoter of ROBO4 detecting several candidate TFBSes, including SP1 and a GA-binding protein (GABP)-binding motifs. In transgenic mice, the GABP site was shown to contribute to ROBO4’s EC expression [62].

Can Okada’s TFBSes be found in ENCODE? This question is answered in S2*A* Fig. Two clusters of TFBSes can be found exactly in the 3-kb region upstream of the TSS on which Okada *et al*. focused (2007). There was a weak SP1 site (QS = 128) but it came from a carcinoma cell-line not ECs. There was no site for GABP. This discrepancy is likely due to the fact that ENCODE did not include GABP or SP1 ChIP-seq on ECs [63] examined by Okada *et al*.

However, ENCODE data suggested a new definition of the architecture of the proximal promoter of ROBO4. The data also facilitated its comparison between ECs and a non-EC cell types (S2 Fig). There was an endothelial GATA-2 TFBS overlapping a strongly-conserved DNA region, multiple CTCF motifs, and two AP-1 canonical DNA motifs together with matching JUN/FOS peaks from ECs. (Note that AP-1 is a hetero-dimmer of Jun and Fos family TFs.) As FOS and JUN are proto-oncogenes implicated in the regulation of cell-cycle, proliferation and tumor progression, the above is congruent with ROBO4’s link to proliferating tumor endothelium [2]. The presence of the GATA-2 TFBS is also easily explained. GATA-2 is highly expressed in ECs [64] and known as a key regulator of EC transcription [65]. GATA-2 is also a hematopoietic marker [66] which is congruent with a hypothesis of the common evolutionary and developmental origins of ECs and blood cells.

Finally, we note that DNase I signal suggested that the promoter was accessible in 66–39% of 125 ENCODE cell lines. Such open chromatin has to attract TFs and indeed many weak non-EC TFBSes overlapped endothelial ones. Such overlapping clustering of TFBSes suggests they form complexes and cooperate. It is tempting to speculate that the weak non-EC TFBSes contribute to endothelial-specificity of ROBO4’s as inhibitors of transcription which can bind the regions of open chromatin when they are not bound by endothelial-specific TFs. However, an extensive analysis of genome-wide trends would be necessary to test this hypothesis and this was not in the scope of the current manuscript.

**Fig 5:**
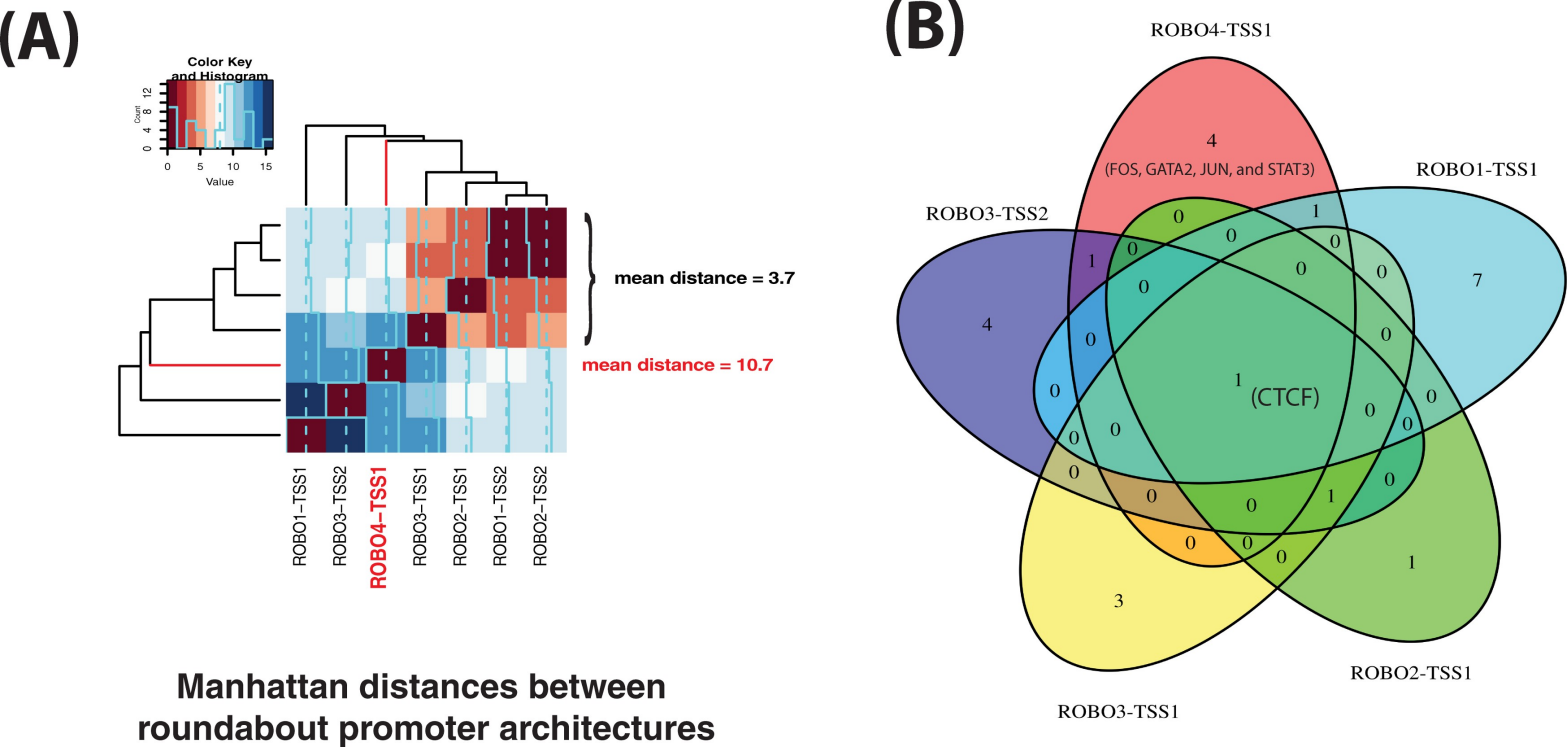
The promoter architecture associated with ROBO4-TSS1 is dissimilar to those of other roundabout TSSes. In this figure, the promoter architecture of ROBO4-TSS1 is compared against the architectures of other roundabouts using the following two approaches: pairwise Manhattan distances and a Venn diagram. In panel (A), a heatmap visualizes Manhattan distances between vectorized promoter architectures of the TSSes. Short distances indicate similarity, long distances indicate divergence. The average distance to ROBO4-TSS1 was 10.7 (N = 6). The distances between similar promoter architectures of ROBO2-TSS1, ROBO2-TSS2, ROBO1-TSS2, and ROBO3-TSS1 (N = 6), which cluster in the heatmap, are significantly shorter (mean 3.7, Wilcoxon *P*-value 0.005). To put these distances in context, we note that the average distance between all roundabout TSSes (N = 21, all pairwise comparisons excluding self-comparisons) equaled 8.67 and was almost twice shorter than the average distance between random pairs of RefSeq promoters (16.259, N = 834,585,940, Wilcoxon *P*-value = 0.03614). Panel (B) shows a Venn diagram for the sets of strong TFBSes in the architectures of ROBO4-TSS1, ROBO1-TSS1, ROBO2-TSS1, ROBO3-TSS1 and ROBO3-TSS2. The five architectures have only one TF binding site in common: CTCF.

The architecture of the promoter of ROBO4 was compared against others in the genome including those of roundabout paralogs. It was found to be unique in the genome, highly unlikely to have been assembled by chance, and dissimilar to those of the architectures of other roundabout promoters (Fig 5A and 5*B*). Moreover, ROBO4-TSS1 had four strong TFBSes that the other roundabout TSSes did not have: FOS, GATA2, JUN, and STAT3. The TSS shares maximally only two strong ENCODE sites with the other roundabout architectures. The above features are suggestive of regulatory neo-functionalization of the ROBO4 promoter. As shown by the simulation, chances are that under the DDC model there would be higher similarity between ROBO4-TSS1 and the most likely candidate paralog promoter architecture: ROBO1-TSS1. Certainty could only come with the knowledge of the ancestral state pre-2R-WGD and such data cannot be available. However, we note ROBO4-TSS1 is not a likely candidate for the DDC model, as endothelial expression could not have been a part of the ancestral pre-2R expression domain.

Could ROBO4 have acquired an entirely new promoter after duplication, down-stream of the original TSS? This is strongly suggested by the fact that the ROBO4 protein is N-terminally truncated in relation to the alignment of other roundabouts (by 186 positions, S2 File). This includes the ROBO1 isoform transcribed from ROBO1-TSS1 (and ROBO1-TSS2 which lies even further upstream). In effect, the first exons of both ROBO1 isoforms have no homology to ROBO4 (note green ‘^’ in Fig 4). Such an evolutionary scenario supports the model of regulatory neo-functionalization rather than the DDC. Since the architecture of this new promoter is complex, it seems improbable that it emerged *de novo*, but no further clues about its origins can be offered here. In any case, ROBO4-TSS1 almost certainly is not homologous to any of the other TSSes of roundabouts.

It is generally recognized that the divergences of expression patterns and protein sequences are the two fundamental mechanisms which generate organismal diversity [67]. In some cases, the two mechanisms closely correlate with each other. In other cases, one of the mechanisms is dominant. It was suggested that morphogens evolve mostly on the level of expression, while genes involved in physiological traits evolve on protein level [68]. In the case of ROBO4, the asymmetric divergence of its expression pattern correlates positively with the asymmetric divergence of its coding sequence. This was first suggested by the relative lengths of the ROBO4 and ROBO1 protein branches in Fig 2. The analysis of evolutionary rates using PAML confirmed that ROBO4 had increased evolutionary rate (Table 6). The ratio of synonymous over non-synonymous substitutions on the ROBO4 branch was increased 5–6 fold in comparison to the background evolutionary rate of 0.11 in the rest of the tree. Moreover, many individual amino-acids in the sequence of ROBO4 (~9%) were indicative of positive selection. Thus, ROBO4 diverged at an accelerated rate both in expression and in protein sequence.

What can be said about the new biological roles and biochemical functions of ROBO4? Are they also neo-functionalizations? It is certain that the biological function of ROBO4 switched from axon guidance to the control of angiogenesis and vascular permeability. Details of this role are still an active area of research reviewed by Roy Bicknell and co-workers [69, 70]. There are two interpretations of the mode of action of ROBO4: as pro-angiogenic or anti-angiogenic. The two interpretations could be reconciled if the effects of ROBO4 signaling were interpreted as context-dependent. For example, ROBO4 signaling could be modulated by other angiogenic factors and their receptors and dependent on the microenvironment and the location of the endothelial cell in the growing capillary.

Naturally, ROBO4 can fulfill its roles while interacting with other roundabout receptors. STRING data (Fig 3) and literature (Table 2) suggest that ROBO4 interacts with ROBO1 which is further supported by ROBO1’s expression in endothelial libraries (albeit at levels 10-fold lower than those of ROBO4). A truncated ROBO3 produced by ROBO3-TSS1 (missing the first 17 coding exons encoding the entire extra-cellular domain) is also a possible but speculative interaction partner. Both the above scenarios conform to a paradigm where a neo-functionalized protein fulfills its function in hetero-dimers with paralogs. The examples of such hetero-dimers include type I and type II TGF-ß receptors which also diversified in the course of 2R-WGD [34]. The heterodimerization seems very likely if the ancestral protein could homodimerize as it had to include a self-binding interface which could simply be re-used for binding a paralog protein. Indeed, robo in the fly can [71] suggesting this was the ancestral state pre-2R-WGD.

Note that the direct interaction between ROBO4 and hSLIT2 (Table 2) that was proposed by Park *et al*. [72] is hotly contested. In an influential study, Suchting *et al*. [73] showed that Robo4 failed to bind Slits. Moreover, Robo4 does not have the critical binding residues necessary for this interaction [74]. Instead, Robo4 was shown to bind with high affinity the Unc-5 netrin receptor B—hUnc5b [75]. The activation of Unc5b stabilizes vasculature and inhibits its permeability by opposing the signaling of the vascular endothelial growth factor—VEGF [75]. Further note that there are four vertebrate paralogs (Unc5a, Unc5b, Unc5c and Unc5d) of a single Drosophila receptor—UNC-5 [76, 77]. This suggests that the family of Unc-5 receptors also diversified through gene duplication.

Interestingly, the transgenic studies of mouse knock-out strains indicated that Robo4, unlike Robo1/2, is not essential for developmental angiogenesis [78, 79]. The reason is probably that increased vascular permeability in Robo4 knock-out mice is compensated by increased levels of plasma renin [75]. Instead, the function of Robo4 is more apparent in non-developmental contexts when normal organismal homeostasis is disrupted, for example under stress or in pathological conditions. Examples include wound healing or the diabetic retina [80]. Thus, this is an interesting example of a compensatory mechanism that does not directly depend on the paralog of Robo4. One is tempted to speculate that in complex vertebrate species, multiple organ systems exert multiple levels of physiological control for critical body functions such as the maintenance of the homeostasis of blood pressure. This challenges the traditional view of neo-functionalization that was so far tested mostly using genomics data from unicellular organisms such as yeasts.

More generally, the STRING database suggests that ROBO4 functions as a network bridge integrating three diverse cellular processes: angiogenesis, axon guidance and filopodia formation. While ROBO4 shares some interaction partners with ROBO1, the ROBO1 paralog is not a signaling bridge and does not have interaction partners in the angiogenesis and filopodia networks. This is in agreement with the predictions made by the neo-functionalization model for the interaction network. What is also interesting about ROBO4’s interaction network is that, apart from roundabouts, several other proteins are 2R-ohnologs (Table 8). Thus, the entire endothelial-specific network emerged through 2R-WGD suggesting the genomic doubling was the key to the emergence of the endothelium and the pressurized circulatory system.

**Table 8:** Gene families of putative 2R-ohnologs in the signaling and regulatory networks of ROBO4. Paralogs were inferred from TreeFam v9 from trees corresponding to family IDs given (www.treefam.org/). In each case, the most recent duplication event was placed at the base of vertebrates by phylogenetic timing. The paralogs listed are descendants of such vertebrate duplication nodes. The ancestral bilaterian gene was inferred on the basis of the fly or worm ortholog. Paralogs directly in the network are highlighted in bold font. Note that many gene members of these gene families are preferentially expressed in ECs, for example: KDR, FLT1, TIE1, TIE2, ROBO4, GATA2.

TreeFam family ID	2R-ohnologs (vertebrate paralogs)	Ancestral bilaterian gene
Receptors		
TF325768	kinase insert domain receptor (KDR); **fms related tyrosine kinase 1 (FLT1);** fms related tyrosine kinase 4 (FLT4);	PDGF- and VEGF-receptor related (Pvr)
TF317568	tyrosine kinase with immunoglobulin like and EGF like domains 1 (TIE1); **TEK Receptor Tyrosine Kinase (TIE2);**	Unknown
TF351053	**roundabout guidance receptor 1 (ROBO1);** roundabout guidance receptor 2 (ROBO2); roundabout guidance receptor 3 (ROBO3); **roundabout guidance receptor 4 (ROBO4);**	roundabout guidance receptor (robo)
Ligands		
TF319554	vascular endothelial growth factor A (VEGFA); vascular endothelial growth factor A (VEGFB); vascular endothelial growth factor A (VEGFC);	PDGF- and VEGF-related factor 1 (Pvf1)
TF336658	angiopoietin 1 (ANGPT1); angiopoietin 2 (ANGPT2); angiopoietin 4 (ANGPT4);	uncertain or missing
TF332887	**slit guidance ligand 1 (SLIT1);** **slit guidance ligand 2 (SLIT2);** **slit guidance ligand 3 (SLIT3);**	secreted glycoprotein Slit (sli)
Transcription factors		
TF315391	GATA binding protein 1 (GATA1); GATA binding protein 2 (GATA2); GATA binding protein 3 (GATA3);	Grain (Grn)
TF106430	CCCTC-binding factor (CTCF); Brother of Regulator of Imprinted Sites (CTCFL);	CTCF
TF318648	signal transducer and activator of transcription 1 (STAT1); signal transducer and activator of transcription 2 (STAT2); signal transducer and activator of transcription 3 (STAT3);	signal transducer and activator of transcription 1 (Sta-1)
TF316127	forkhead box A1 (FOXA1); forkhead box A2 (FOXA2); forkhead box A3 (FOXA3).	Forkhead (fkh)

## Conclusions

Vertebrate roundabouts are arranged in two clusters (ROBO2-ROBO1 and ROBO3-ROBO4). The expression of ROBO4 in endothelial cells is a clear neo-functionalization on the level of gene expression. We can speculate that ROBO4 neo-functionalized to its endothelial-specific expression because of the powerful adaptive advantage conferred by the pressurized circulatory system which ROBO4 helped to shape and which emerged through 2R-WGD. In terms of the protein interaction network, ROBO4 functions as a bridge integrating several functional sub-networks of the mammalian cell, with a change of ligand to the hUnc5b receptor which is also expressed in endothelial cells [81]. Thus, ROBO4 neo-functionalized on several levels: (i) on the level of the expression pattern, (ii) on the level of the biochemical function—in particular with respect to its ligand, and (iii) on the level of the biological process. ROBO4 is a very clear and illustrative example of the emergence of a vertebrate molecular evolutionary novelty and a vertebrate-specific signaling network through neo-functionalization coupled with the emergence of ECs. The high rate of the preservation of paralogs following 2R-WGD may be explained by the emergence of novel cell types with corresponding cell-specific regulatory networks. Here, it is important to realize that the DDC model of duplicate retention was originally proposed to explain the high rate of duplicated retention following animal WGDs [15] which Force *et al*. thought could not have been explained by neo-functionalization. In this context, our results suggest that the emergence of novel cell types, such as the endothelium, could be a contributing factor that was neglected but which works surprisingly well with the classical model of duplicate retention through neo-functionalization, at least after 2R-WGD.

## Methods

### The alignment of roundabouts and the building of TreeBeST phylogenomic trees

The amino-acid sequences of vertebrate roundabouts were obtained from GenBank (accessions given in Table 9). The protein sequences were aligned using MUltiple Sequence Comparison by Log-Expectation (MUSCLE) v3.8.31 [82, 83], a very fast algorithm which produces alignments at least as accurate as its competitors (CLUSTALW, T-Coffee, or MAFFT). The corresponding nucleotide sequences of the CDSes were aligned using this protein alignment as guide with the Python script revtrans.py v1.4 [84]. This nucleotide-level alignment of roundabouts was then used as the input for the TreeBeST (gene Tree Building guided by Species Tree) meta-builder of phylogenetic trees (TreeBeST version 1.9.2) and the PAML analysis. TreeBeST merges trees derived from several algorithms such as neighbor-joining and Phyml [85]. TreeBeST calculated a consensus tree outputted in the New Hampshire eXtended (NHX) format [86]. This TreeBeST NHX-file for roundabouts is shown in the S3 File. In the NHX format, additional tags are used to annotate internal nodes as either duplication or speciation nodes together with a species tag assigned through phylogenetic timing, that is on the basis of the distribution of orthologs in extant taxa.

**Table 9:** The accessions of the sequences of roundabouts.

Species	Gene	Protein accession	Amino-acids	Coding sequence.
Human	ROBO1	NP_002932	1651	CCDS54611
	ROBO2	NP_001276969	1443	NM_00129004, 644–4975 bps
	ROBO3	NP_071765	1386	CCDS44755
	ROBO4	NP_061928	1007	CCDS8455
Mouse	Robo1	NP_062286	1612	CCDS37376
	Robo2	NP_780758	1508	CCDS49886
	Robo3	NP_001158239	1402	CCDS52770
	Robo4	NP_001296319	1022	CCDS80976
-Rat	Robo1	NP_071524	1651	NM_022188, 1–4956 bps
	Robo2	NP_115289	1512	NM_032106, 459–4997 bps
	Robo3	NP_001101605	1305	NM_001108135, 352–4269 bps
	Robo4	NP_852040	961	NM_181375, 1–2886 bps
Zebrafish	Robo1	NP_001296753	1646	NM_001309824, 699–5639 bps
	Robo2	NP_571708	1513	NM_131633, 168–4709 bps
	Robo3	NP_001315345	1419	NM_001328416, 355–4614 bps
	Robo4	XP_689255	1134	XM_684163, 647–4051 bps
Chicken	Robo1	XP_015153996	1573	XM_015298510, 326–5047 bps
	Robo2	XP_015154089	1516	XM_015298603, 419–4969 bps
	Robo3	XP_015153567	1232	XM_015298081, 196–3894 bps
	Robo4	XP_015153568	1064	XM_015298082, 335–3529 bps
Fly	Robo1	NP_476899	1395	NM_057551, 176–4363 bps
	Robo2	NP_536792	1463	NM_080531, 289–4680 bps
	Robo3	NP_001259866	1342	NM_001272937, 475–4503 bps
Worm	Sax-3	NP_001024990	1273	NM_001029819, 1–3822 bps

### The inference and timing of gene duplications

Gene duplications for roundabouts were inferred by TreeBeST using a speciation-duplication inference algorithm that reconciles the species tree with the gene tree. However, duplication nodes without species support (that is with the SIS tag set to the value of zero) were removed. Such duplication nodes are never genuine. The method used by TreeBeST requires the assumption of the known species tree; we used the species tree provided as the part of the TreeBeST package version 1.9.2 (S4 File). This species tree assumes the Coelomata hypothesis [87] for the evolution of the animal kingdom. We note that the topology of the TreeBest tree in Fig 2 is in agreement with the phylogenetic tree of roundabouts derived directly from the TreeFam v9 database (accessed online, family accession number: TF351053, see S4 Fig).

### The conservation of a two-gene cluster in vertebrates

To estimate the probability of the conservation of a two-gene cluster analogous to ROBO3-ROBO4 we analyzed ENSEMBL orthologs. The orthologs of human genes were downloaded from the Ensembl Genes 89 BioMart [88] to a local MySQL database. Only one-to-one orthologs were selected from the following species: *Mus musculus* (16,796), *Felis catus* (15,491), *C*. *lupus familiaris* (15,867), *Bos Taurus* (16,351), *Equus caballus* (16,122), *X*. *tropicalis* (12,024) and *Danio rerio* (9,908). The lesser number of one-to-one orthologs in *Danio rerio* is probably a consequence of a fish-specific whole genome duplication [89]. In the human genome, 50,498 gene pairs with the positions of gene starts spaced less than 100,000 bps were identified. 1051 of these gene pairs had a one-to-one ortholog in all the seven species. The human gene pair was classified as conserved in a given species if both orthologs were clustered on the same chromosome within 200,000 bps. The latter stages of the analysis were performed using custom written Python 3 and R scripts.

### MrBayes trees

To test the robustness of the phylogeny of roundabouts, we calculated Mr. Bayes consensus trees (50% majority rule) with varying analysis parameters. All trees were calculated for the amino-acid sequences of either: (W) whole protein, (E) extra-cellular portion, or (I) the intracellular portion of roundabouts. Trees were calculated with the *aamodelpr* set to *mixed* (where ten different amino-acid substitution models are sampled according to their posterior probabilities). The rates parameter sets the model for the variation of evolutionary rates among amino-acid sites: *equal* — no variation; *gamma* — the rate of a site is drawn from a gamma distribution; *invgamma* — a fraction of sites is invariable while others come from a gamma distribution. Those trees were shown in the S5 File and displayed as text-trees with Mesquite version 3.2.

### F5 expression profiles of individual TSSes and genomic sub-loci

F5 used CAGE technology to construct an atlas of gene expression in the human genome at a single-base resolution level [90]. F5 human included libraries for 179 tissues, 513 primary cell isolates, and 260 libraries prepared from cancer cell-lines. We analyzed FANTOM5 data using either the ZENBU browser or R after downloading work-package 4 (WP4) expression tables.

Fig 4 and S1 Fig were generated using the ZENBU browser (http://fantom.gsc.riken.jp/zenbu/). ZENBU was developed specifically to visualize F5 data in genomic coordinates [91]. All data was mapped on the hg19 human assembly or the mouse mm9 assembly. Phase 1 and phase 2 CAGE tracks were pooled, *rle* normalized, and filtered (such that clusters of 3 or more tags per library are shown). For expression data, either rank-sum enrichment was calculated, or the number of tags normalized per million tags in the library. Rank-sum enrichment was calculated by a Wilcoxon-mann-whitney rank-sum enrichment algorithm. The algorithm returned a z-score that was proportionate to the enrichment of expression in the sets of samples grouped in different ontologies.

To calculate the relative expression of human roundabout TSSes in endothelial versus non-endothelial cells, 28 endothelial F5 libraries were identified among the total of 513 primary cell F5 libraries. The endothelial libraries included aortic, arterial, micro-vascular, thoracic, vein, umbilical vein, hepatic sinusoidal, glomerular, and lymphatic endothelial cells (with biological and technical replicates). In this sub-analysis, F5 tissues and cancer cell lines were not included as: (1) bulk tissues contain an infiltrating fraction of endothelial cells, (2) tissue-specificity in immortalized and transformed cancer cell lines can be altered. Preferential endothelial expression was the ratio of average expression in the endothelial over the non-endothelial subset.

### Co-expression of roundabouts

To test for the co-expression of ROBO4 with other roundabouts (Table 4 and Fig 4*C*), Pearson product moment correlation coefficients (PCCes) and asymptotic *P*-values were calculated with the core R function *cor*.*test()*. The parameter *method* set to “pearson”. PCC was used because we previously found it works well for tissue-specific genes and in the context of the analysis of gene duplicates [11]. In our hands, PCC was more sensitive to the signal of the co-expression of paralogs than non-parametric alternatives such as Spearman’s *rho* and Kendall’s *tau*.

However, the *P*-value accompanying the PCC of a pair of co-expressed genes may not be reliable as *cor*.*test()* uses a t-distribution assuming that samples are normally distributed. Therefore, alternative *P*-values were calculated using sampled randomization. In this approach, the assignments of expression values to all RefSeq transcripts were permuted for each sample of expression. After each of the 1000 permutations, a sample of 1,000 RefSeqs was selected at random and PCCes were calculated for all possible pairs in the subset excluding self-comparisons. The distribution of the PCCes obtained through the above permutations defined an empirical cumulative distribution function. Percentiles of this distribution calculated for the PCCes from the comparisons of roundabouts defined randomization *P*-values displayed in Table 4.

### The STRING database of the functional interactions of proteins

STRING is a bioinformatics resource where protein interactions are inferred from five lines of evidence: (1) genomic context, (2) high-throughput experiments, (3) co-expression, (4) text mining, and (5) other databases such as BioGRID [92]. Version 10.0 of the database was used (with medium confidence cut-off: STRING score of more than 0.4). We note that STRING interactions are functional rather then physical.

### The prediction of Pfam protein domains with *hmmscan*

The program *hmmscan* from the HMMER package v3.1*b2* was used to test roundabout protein sequences against the database of 16,306 hidden Markov models of protein domains from the Pfam database release 30. *E*-value, that is the expected number of random hits of equal strength, was calculated by the program *hmmscan* from the HMMER v3.1 package [93]. Only *hmmscan* hits (S6 File) with the *E*-value lesser than 10e-10 were reported. We note that *hmmscan* may report multiple domain hits in the region (for example, from alternative definitions of the immunoglobulin domain in the database). For this reason, only a single lowest scoring domain was reported for each region of the protein query in Table 10 (*i*.*e*. the reported domains are non-overlapping).

**Table 10:** The protein domains of roundabouts.

Gene ID	Domain-name	E-value	START*	END*
ROBO1	Ig_3	1.5e-84	67	151
	I-set	2.3e-90	172	257
	Ig_3	1.5e-84	261	334
	Ig_3	1.5e-84	350	432
	Ig_3	1.5e-84	454	528
	V-set	3.5e-28	504	537
	fn3	8e-35	562	646
	V-set	3.5e-28	675	721
	fn3	8e-35	778	864
	Ig_2	5.6e-52	944	961
	C2-set_2	5.2e-18	1253	1269
ROBO2	Ig_5	3.2e-11	104	114
	I-set	1.4e-88	135	221
	I-set	1.4e-88	225	310
	Ig_3	1.2e-82	317	397
	Ig_3	1.2e-82	421	495
	fn3	1.5e-35	528	611
	Ig_3	1.2e-82	651	669
	fn3	1.5e-35	743	830
ROBO3	V-set	5.9e-30	137	161
	I-set	1.1e-82	168	249
	I-set	1.1e-82	258	343
	Ig_3	3.3e-78	346	426
	Ig_3	3.3e-78	450	524
	fn3	2.1e-31	558	641
	I-set	1.1e-82	682	694
	fn3	2.1e-31	782	857
	C2-set_2	1.4e-15	1143	1162
ROBO4	Ig	1.5e-13	107	124
	I-set	3.6e-24	138	220
	fn3	1.5e-10	264	333
	fn3	1.5e-10	350	432
	Ig_2	6.7e-17	985	1005

NOTE: I-set (PF07679.14), V-set (PF07686.15), C2-set_2 (PF08205.10), ig (PF00047.23), Ig_2 (PF13895.4), and Ig_3 (PF13927.4) are sub-types of immunoglobulin domains; fn3 — fibronectin type III domain (PF00041.19). E-value, that is the expected number of random hits of equal strength, was calculated by the program *hmmscan* from the HMMER v3.1 package.

The lesser number of protein-binding domains in the ROBO4 extra-cellular domain is the consequence of the loss of coding exons. Human ROBO4 has 18 exons, while ROBOs 1/2/3 have 29, 27 and 28, respectively. (The numbers of exons were derived from the UCSC Genome Browser on the hg38 assembly, gene models for ROBO4 (transcript ENST00000306534.7), ROBO1 (ENST00000467549.5), ROBO2 (ENST00000332191.12) and ROBO3 (ENST00000397801.5).)

* START and END positions are given in the coordinates of the amino-acid sequence of the roundabout.

### The promoter architectures of roundabouts

The ENCODE dataset included data for 161 TFs and 91 human cell types under various treatment conditions [94]. This included assays on primary human endothelial cells: HUVECSs for CTCF, FOS, GATA2, EZH2, JUN, MAX, and MYC transcription factors. These ENCODE TFBSes, mapped on the February 2009 human reference sequence (GRCh37), were accessed using the UCSC genome browser (the archive for the human genome assembly GRCh37/hg19 at genome.ucsc.edu).

The ENCODE dataset was also downloaded for offline analysis as a BED file [95]. The BED file was compared against 6kb-wide promoter targets of: (i) roundabouts in hg19 coordinates (Table 11), (ii) RefSeq reference transcripts. The comparison was performed using intersectBed (bedtools) with one file being the ENCODE BED file; the other file was a target file prepared using a Python script from Genome Browser-downloaded hg19-mapped RefSeq transcripts (40,856 transcripts). The results of intersectBed were parsed using an R script. The offline analyses included the determination of promoter architectures, promoter probabilities and pairwise distances.

**Table 11:** The locations of the promoters of roundabouts in the human genome (hg19 coordinates) and corresponding regions in mouse (mm9). For each promoter, maximal expression (ME) for both human and mouse is given in square brackets.

Promoter ID	Closest RefSeq	ENSEMBL gene ID	Location in the human genome and maximal expression [ME]	Top mouse BLAT hit: location (score) and [ME] Conserved?
ROBO4-TSS1	NM_019055	ENSG00000154133	chr11:124764760–124770760 **[979]**	chr9:37208261–37212629 (**904**) **[305] YES**
ROBO3-TSS2	NM_022370	ENSG00000154134	chr11:124732300–124738300 **[28]**	chr9:37239055–37242808 (**718**) **[34] YES**
ROBO3-TSS1	NM_022370	ENSG00000154134	chr11:124743700–124749700 **[41]**	chr9:37225070–37231494 (**1717**) **[3] NO**
ROBO2-TSS2	NM_001128929	ENSG00000185008	chr3:75952845–75958845 **[8]**	chr16:75420561–75421064 (**97**) **[0] NO**
ROBO2-TSS1	NM_002942	ENSG00000185008	chr3:77086294–77092294 **[341]**	chr16:74409016–74413502 (**1146**) **[166] YES**
ROBO1-TSS1	NM_133631	ENSG00000169855	chr3:79065600–79071600 **[191]**	chr16:72662054–72666244 (**1232**) **[115] YES**
ROBO1-TSS2	NM_002841	ENSG00000169855	chr3:79814200–79820200 **[135]**	chr16:72024757–72029898 (**916**) **[81] YES**

Importantly, ENCODE TF binding sites (TFBSes) are assigned a quality score (QS) which can be used to divide them into strong (QS > 500) and weak sites (QS ≤ 500). Unless otherwise stated, only the strong ENCODE sites were used in the analysis.

The distribution of the sizes of promoter architectures (that is the cardinalities of the corresponding sets) is not normal (mean = 9.2, median = 4, variance = 139.8, skewness = 1.775, kurtosis = 6.859, min = 0, max = 106).

### The divergence between the architectures of the promoters of roundabouts

To estimate their divergence, promoter architectures were first encoded as vectors in 161 dimensions. The number of dimensions corresponded to the number of TFs assayed by ENCODE. Next, Manhattan distances between each pair of vectorized promoter architectures were calculated (Fig 5*A*). To put roundabout distances in context, they were compared with promoters of all RefSeq transcripts (see Fig 5 legend). Note, that the aim was to estimate the divergence of promoter architectures—the repertoires of interacting TFBSes, rather than between the nucleotide sequences of promoter regions which are too diverged for a meaningful comparison.

The choice of the metrics should be defended. Manhattan rather than Euclidean distances were used as the latter have counter-intuitive properties in multidimensional spaces [96]. Another alternative would be to use a measure of distance between sets such as the Jaccard index (JI) which is the ratio of two sets’ intersection over the union [97]. However, JI could return large distances even for single-element differences between small sets. Conceptualize a pair of two-elements sets which have one element in common: JI equals 1/3 which is 1/3 maximum, while manhattan distance equals 1 which is 1/161 maximum. Therefore, manhattan distance, which can be used because the repertoire of TFs is known, is preferable for the current analysis as it has a tendency to return small values for small promoter architectures.

### The probabilities of the promoter architectures of roundabouts

Some promoter architectures may be more frequent than others simply because the probability of the occurrence of respective TFBSes in promoters is high. Other architectures may be rare; the random chance of finding given arrangement of TFBSes in the same proximal promoter may be extremely low. Rare promoter architectures may be speculatively interpreted as more informative.

To estimate the degree of non-randomness associated with the promoter architectures of roundabouts, we calculated joint probability of each of them as an event in the multivariate probability space composed of 161 discrete random variables (*TFBS*_*k*_,*k* = 1,…,161). Thus, each random variable *TFBS*_*k*_ corresponded to counts of individual TFBSes from the merged ENCODE data. The expected probability of a given binarized promoter architecture (P_E_) with *n*-TFs was calculated assuming independence between the sites and was a product of marginal probabilities of all 161 ENCODE TFBSes. P_E_ could be expressed in the following closed-form:

$$P_{E}=\prod_{k=1}^{n}{P(TFBS}_{k}=1)\times \prod_{k=n+1}^{161}{P(TFBS}_{k}=0),$$

where TFBS_k_ equaled one for TFs which were present in the promoter architecture (that is TFs with indices from 1 to n) and zero for TFs which were not present (that is TFs with indices from n+1 to 161).

The observed probability of a given promoter architecture (P_O_) was simply a ratio of transcript which carried such an architecture in the total population of RefSeq transcripts.

To calculate both P_E_ and P_O_, the vectors representing promoter architectures were converted to binary, that is 0 or 1 (representing the presence or absence of a TF binding site). The ratio of P_O_/P_E_ is the measure of enrichment of a given combination of TFs in real data versus random expectation. Again, to put the values obtained for roundabouts in context, we compared them against the distributions of P_E_, P_O_ and P_O_/P_E_ for all RefSeq promoter architectures (Table B in S1 File).

### Homology modeling

The homology model used for illustrative purposes in conceptual Fig 1 was generated by the Swiss Model server (swissmodel.expasy.org). We used the template of receptor-type tyrosine-protein phosphatase delta with 28% identity over 309 amino-acids located in the N-terminal part of the isoform 2 of ROBO4 (GenBank NP_001288017.1).

### The calculations of the evolutionary rate

The *codeml* program from the version 4.8 of the PAML package was used [98]. *Codeml* uses maximum likelihood estimation of parameters: evolutionary rates and proportions of sites in different classes. The codons of human, mouse and *Drosophila* roundabouts were analyzed (this subset of the data was called a small data set). The following Newick format tree assuming 2R-WGD phylogeny of roundabouts was used:

*“(dmRobo1*, *( (hsROBO4*, *mmRobo4 ) #1*, *(hsROBO3*, *mmRobo3 ) )*, *( (hsROBO1*, *mmRobo1 )*, *(hsROBO2*, *mmRobo2 ) ) );”*. This tree intentionally select the branch of ROBO4 *before* the divergence with the murine ortholog as foreground. Any period of accelerated evolution or positive selection would be expected immediately after 2R-WGD rather than during the diversification of mammals. 2R-WGD tool place more than 450 million years ago (MYA). The evolutionary split between human and mouse occurred 65–75 MYA [99].

The alternative models of evolution were evaluated using the likelihood-ratio test and the chi-square test of significance following standard protocols [57, 100]. To statistically compare a pair of models, first twice the difference in their likelihoods was calculated. Then a percentile was taken of the chi-squared distribution with the *d*.*f*. equal to difference in *d*.*f*. between the two models. If the percentile was lower than the critical value of 5%, the difference was statistically significant at the *P*-value of 0.05.

Bayes empirical Bayes, otherwise known as the BEB test [101] was used to identify individual positively selected sites in models which allowed the rate of evolution to vary both between the branches and between the sites. Sites with the BEB probability score higher than 0.95 were interpreted as positively selected. The BEB test is a refinement over the previously used naïve Bayes test as it controls for sampling errors in small sets of sequences.

### TSS conservation in mouse

The conservation in mouse of each human roundabout TSS was assessed. For each 6kb promoter region in the human genome linked with a roundabout TSS, genomic nucleotide sequence was retrieved and used as a BLAT search query against the mouse assembly (mm9). The location and score of the top mouse genomic hit is given in Table 11 together with its ME in F5 mouse data. If the ME was above 10 TPM (F5’s cutoff for a gene to be regarded as expressed), the promoter and the associated TSS were regarded as conserved.

### Simulation models

Two types of simulation models were constructed. In the first model (labeled independent-TFs), the presumed ancestral architecture was the union of the sets of TFs bound by both paralog promoters. TFBSes were treated as independent sites with the same probability of the loss. In the second model (labeled clustered-TFs), the ancestral architecture was a 6kb simulated DNA region with all the TFBSes of both paralogs (preserving the coordinates of the locations of the original TFBSes in paralogs). TFBS losses were simulated as cumulative cycles of random deletions of 100 basepair fragments of the DNA of the simulated ancestral promoter. Thus, TFBSes were not independent but clustered as in the original paralog promoters. In both the above simulation models, TFBS deletions in the simulated ancestral architecture were repeated until the algorithm arrived at a pair of promoter architectures of cardinalities equal or lesser than the cardinalities of the input paralog promoters. After each random run, the number of TFBSes in common between resulting paralogs was established by set intersection on their promoter architectures and compared with the observed value. The *P*-value was the fraction of randomization runs where the cardinality of the intersection of the reduced promoters was equal or lesser than the observed value. The runs were repeated 10,000 times. The simulation was implemented in R.

## Supporting information

S1 Fig Vertebrate roundabouts cluster in the genome and this genomic arrangement is conserved. Vertebrate roundabouts are located in two tail-to-tail clusters that are conserved (Table 1). In contrast, invertebrate roundabouts have variable arrangements and do not necessarily cluster. The ancestral bilaterian roundabout receptor was most likely advantageous for the sculpting of the complex bilateral nervous systems, necessary for movement and active search for food. Interestingly, there is no roundabout receptor in either *C*. *savignyi* or *C*. *intestinalis*. This is probably because the roundabout receptor evolved in bilaterians to sculpt their complex nervous systems necessary for movement and active search for food. As tunicates reverted to a stationary lifestyle, roundabouts became dispensable. Sea lamprey (*Petromyzon marinus*) has ROBO3 and ROBO4 orthologs, but they are located on short contigs which does not allow for the evaluation of synteny. Note that the tree is not to scale. The topology reflects the Ecdysozoa hypothesis [109].
(PDF)

S2 Fig The architecture of the proximal promoter of ROBO4: *In vivo* ENCODE TFBSes from HUVEC ECs. In panel (A), a window of 6 kbps around the TSS of ROBO4 was visualized online using the UCSC Genome Browser (archive for the human genome assembly hg19 accessed at http://genome.ucsc.edu/). For the TF track, shades of grey signify the QS-score of the TF binding site (the darker the hue the higher the QS). Note that there are no binding sites in the three kilobases downstream of the TSS (only polymerase type II sites—marked as Pol2). ChIP-seq peaks from HUVECs, which are the most significant for endothelial expression, are highlighted in red. There are two clear clusters of TFBSes: proximal and distal, with a CTCF site between them. Both these clusters have AP1 DNA binding motifs (highlighted as green bars) for the AP-1 hetero-dimer (consisting of JUN and FOS). There are also three CTCF-binding canonical DNA motifs and multiple CTCF ChIP-seq peaks (but only two out of four in HUVECs). Below the TFBS track, there is a wiggle-track with base-wise phyloP 100-vertebrate-genomes conservation scores [110, 111]. Further below, there is a track with DNase I hypersensitive areas assayed in 125 ENCODE cell types (which indicate open, transcriptionally-accessible chromatin). The number to the left of each DNase-box indicates the number of cell types in which it was present. The darkness of each box is proportional to the maximal score in any of the cell types.
(PDF)

S3 Fig Other TSSes of roundabouts. This figure shows additional roundabout promoters shown in the layout analogous to the S2 Fig.
(PDF)

S4 Fig The TreeFam v9 tree for the roundabout family. The figure shows a phylogenetic tree for a TreeFam family with accession number TF351053.
(PDF)

S1 File Supplementary results. This file contains a number of supplementary results: (1) on the conservation of roundabout clusters, (2) an alternative evolutionary model for the emergence of the roundabout cluster, (3) an analysis of expression patterns in genomic sub-regions neighboring the two roundabout clusters, (4) the simulation tests of the DDC model, and (5) the analysis of promoter probabilities. The file also contains two supplementary tables. The first table is devoted to the analysis of expression profiles in the genomic locus of ROBO4 (Table A). The second table is devoted to the probabilistic analysis of the promoter architectures the TSSes associated with roundabouts (Table B).
(DOCX)

S2 File Roundabout alignment. This file contains the alignment of the sequences of roundabouts.
(TXT)

S3 File TreeBeST tree. This file contains the original TreeBeST NHX-file for the phylogenetic tree of roundabouts.
(NHX)

S4 File Species tree. Default TreeBeST package v1.9.2 species tree.
(TXT)

S5 File MrBayes trees. This file contains Mr. Bayes consensus trees with varying analysis parameters.
(DOCX)

S6 File Protein domains. This file contains a list of *hmmscan* hits in the protein sequences of roundabouts.
(TXT)

## Abbreviations

2R-WGD: two rounds of WGD transcription factors
Abl: *D*. *melanogaster* Abl tyrosine kinase
ARHGAP1: Rho GTPase activating protein
ARHGAP39: Rho GTPase activating protein
BEB: Bayes empirical Bayes
BED: browser extensible data
CAGE: cap analysis of gene expression
CDH5: vascular endothelial cadherin
CNS: central nervous system
comm: *D*. *melanogaster* commissureless
CTCF: CCCTC-binding factor
d.f.: degrees of freedom
DDC: duplication degeneration complementation
dlp: *D*. *melanogaster* dally-like
ECs: endothelial cells
ena: *D*. *melanogaster* enabled
ENAH: enabled homolog
ENSEMBL: ENSEMBL genome browser
Eph: *D*. *melanogaster* Eph receptor tyrosine kinase
F5: FANTOM5
FN1: fibronectin 1
Fos: Proto-Oncogene
GABP: GA-binding protein
GATA1: GATA binding protein 1
GATA2: GATA binding protein 2
GATA2: GATA binding protein 2
hENAH: human enabled homolog
hFLT1 (VEGFR1: human Fms related tyrosine kinase 1
hPPARG: human peroxisome proliferator activated receptor gamma
hRXRA: human retinoid X receptor alpha
hSLIT2: human Slit guidance ligand 2
hTHRB: human thyroid hormone receptor beta
hUnc5b: human Unc-5 Netrin Receptor B
HUVECs: human umbilical vein endothelial cells
JI: Jaccard index
JUN: Jun proto-oncogene
lea: *D*. *melanogaster* leak
LRT: likelihood ratio test
ME: maximal expression
ME: maximal expression
mSlit3: mouse Slit guidance ligand 3
MYA: million years ago
NetA: *D*. *melanogaster* netrin A
NetB: *D*. *melanogaster* netrin B
NHX: New Hampshire eXtended format
PCC: Pearson product moment correlation coefficients
PLXND1: plexin D1
PXN: Paxillin
QS: quality score
R1: the first round of 2R-WGD
R2: the second round of 2R-WGD
ROBO 1–4: vertebrate roundabouts 1–4
robo3: *D*. *melanogaster* roundabout 3
ROBO4: Magic roundabout
Saos-2: osteosarcoma cell line
sli: *D*. *melanogaster* slit
SLIT1-3: vertebrate Slit guidance ligands 1–3
STRING: the STRING database of protein interactions
TEK: the TEK angiopoietin receptor
TFBSes: TF binding sites
TGF-ß: transforming growth factor beta
TPM: tags per million
TreeBeST: TreeBeST compound heuristic tree builder
TreeFam: tree families database
TSS: transcription start site
WASL: Wiskott-Aldrich syndrome-like
WGD: whole genome duplication
WIPF1: WAS/WASL interacting protein family member 1
ZENBU: ZENBU expression browser from F5
