# Correction: Magic roundabout is an endothelial-specific ohnolog of ROBO1 which neo-functionalized to an essential new role in angiogenesis

**DOI:** 10.1371/journal.pone.0230384

**Published:** 2020-03-06

**Authors:** Lukasz Huminiecki

There are errors in the Funding statement. The correct Funding statement is as follows: LH was funded to perform this work by the National Science Centre, Poland, grant POLONEZ 2 (grant number 2016/21/P/NZ2/03926). This project has received funding from the European Union’s Horizon 2020 research and innovation programme under the Marie Skłodowska-Curie grant agreement No. 665778. The funders had no role in study design, data collection and analysis, decision to publish, or preparation of the manuscript.
