# Supplementary material for: Magic roundabout is an endothelial-specific ohnolog of ROBO1 which neo-functionalized to an essential new role in angiogenesis
Source: PLoS One. 2019 Feb 25;14(2):e0208952. doi: 10.1371/journal.pone.0208952 (PMC6389290; doi:10.1371/journal.pone.0208952)
Supplement: S1 Fig — Vertebrate roundabouts are located in two tail-to-tail clusters that are conserved (Table 1). In contrast, invertebrate roundabouts have variable arrangements and do not necessarily cluster. The ancestral bilaterian roundabout receptor was most likely advantageous for the sculpting of the complex bilateral nervous systems, necessary for movement and active search for food. Interestingly, there is no roundabout receptor in either C. savignyi or C. intestinalis. This is probably because the roundabout receptor evolved in bilaterians to sculpt their complex nervous systems necessary for movement and active search for food. As tunicates reverted to a stationary lifestyle, roundabouts became dispensable. Sea lamprey (Petromyzon marinus) has ROBO3 and ROBO4 orthologs, but they are located on short contigs which does not allow for the evaluation of synteny. Note that the tree is not to scale. The topology reflects the Ecdysozoa hypothesis [109]. (PDF) [file pone.0208952.s001.pdf]

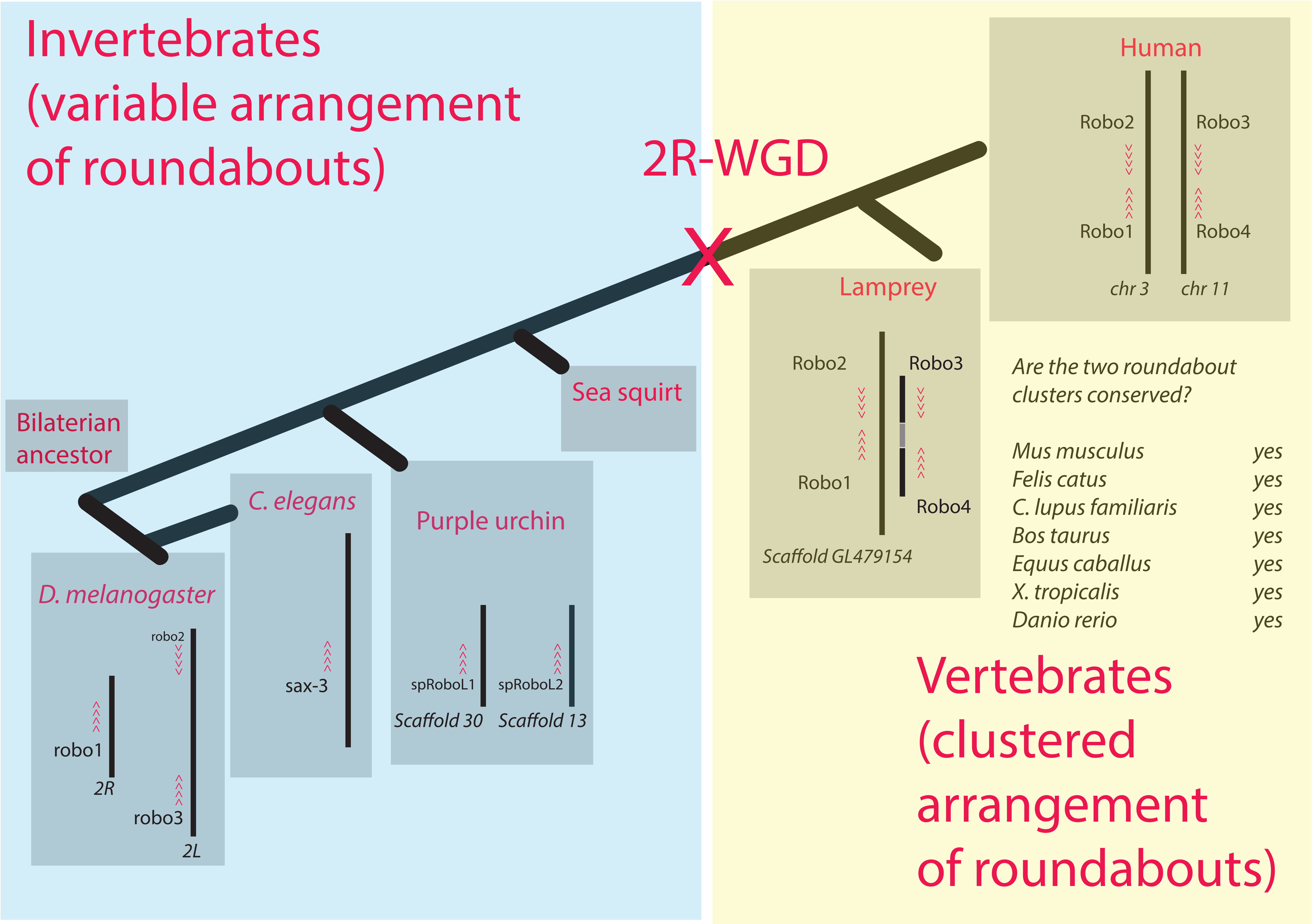
Invertebrates
(variable arrangement
of roundabouts)
2R-WGD
Human
Robo2
Robo3
Robo1
Robo4
chr 3
chr 11
Lamprey
Robo2
Robo3
Robo1
Robo4
Scaffold GL479154
Sea squirt
Bilaterian ancestor
C. elegans
Purple urchin
D. melanogaster
robo2
robo1
2R
robo3
2L
sax-3
spRoboL1
spRoboL2
Scaffold 30
Scaffold 13
Are the two roundabout clusters conserved?
Mus musculus yes
Felis catus yes
C. lupus familiaris yes
Bos taurus yes
Equus caballus yes
X. tropicalis yes
Danio rerio yes
Vertebrates
(clustered
arrangement
of roundabouts)
