# Supplementary figures and images for: Magic roundabout is an endothelial-specific ohnolog of ROBO1 which neo-functionalized to an essential new role in angiogenesis
Source: PLoS One. 2019 Feb 25;14(2):e0208952. doi: 10.1371/journal.pone.0208952 (PMC6389290; doi:10.1371/journal.pone.0208952)

(A) ROBO4-TSS1

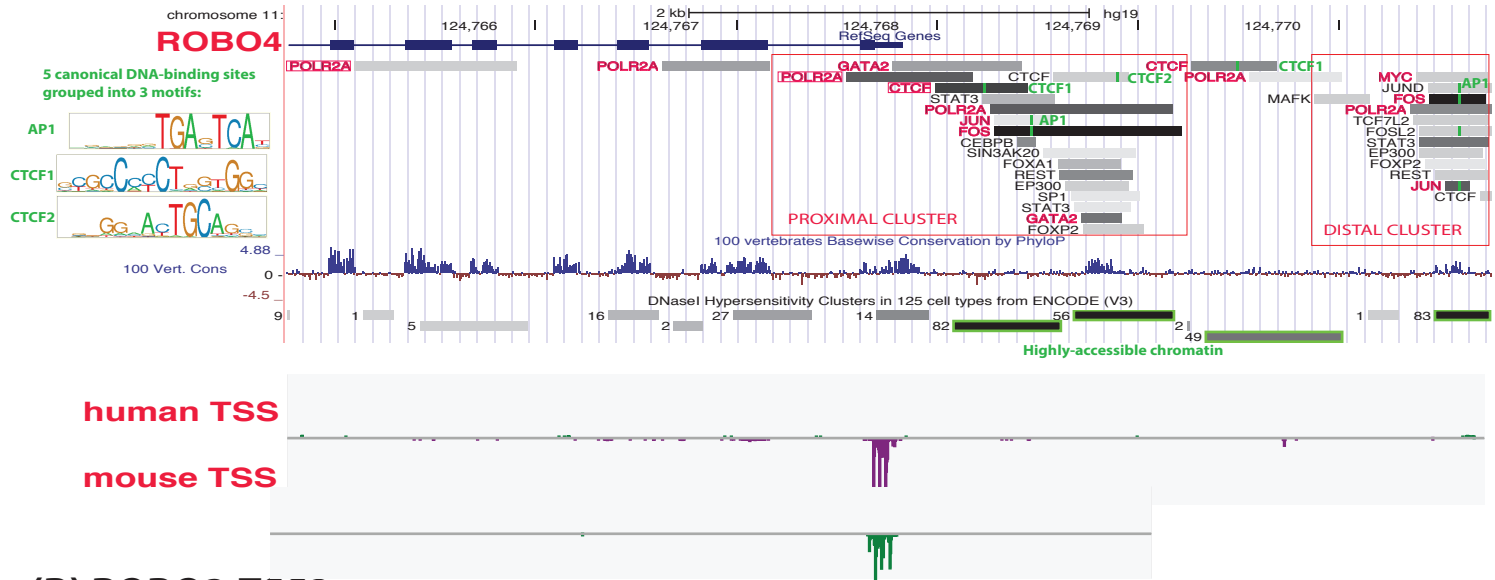

(B) ROBO3-TSS2

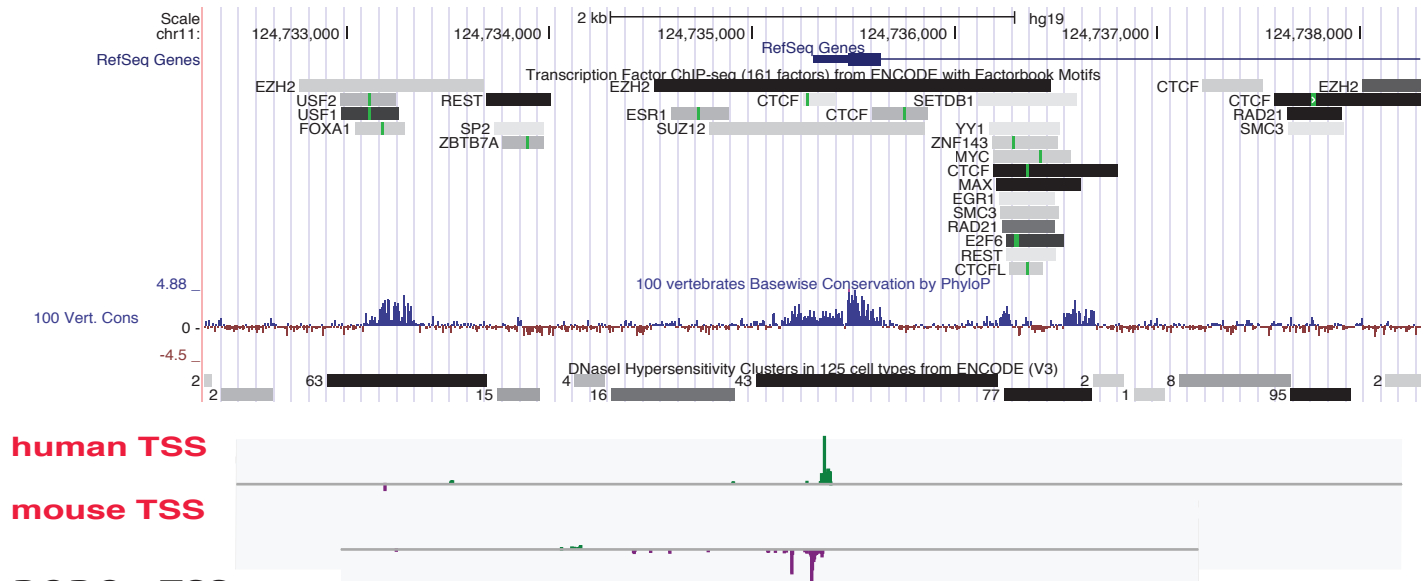

(C) ROBO1-TSS1

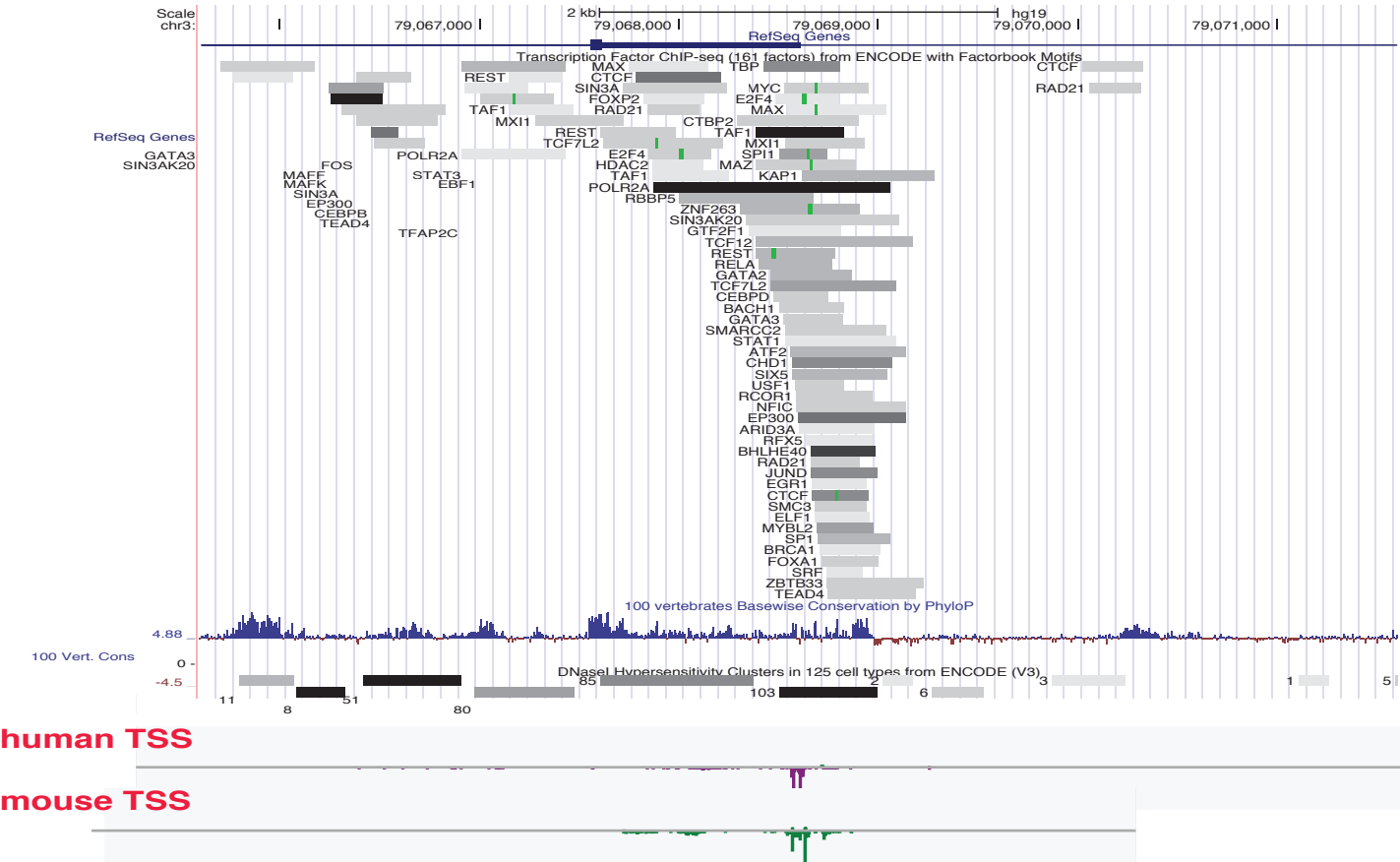

Supplement: S2 Fig — In panel (A), a window of 6 kbps around the TSS of ROBO4 was visualized online using the UCSC Genome Browser (archive for the human genome assembly hg19 accessed at http://genome.ucsc.edu/). For the TF track, shades of grey signify the QS-score of the TF binding site (the darker the hue the higher the QS). Note that there are no binding sites in the three kilobases downstream of the TSS (only polymerase type II sites—marked as Pol2). ChIP-seq peaks from HUVECs, which are the most significant for endothelial expression, are highlighted in red. There are two clear clusters of TFBSes: proximal and distal, with a CTCF site between them. Both these clusters have AP1 DNA binding motifs (highlighted as green bars) for the AP-1 hetero-dimer (consisting of JUN and FOS). There are also three CTCF-binding canonical DNA motifs and multiple CTCF ChIP-seq peaks (but only two out of four in HUVECs). Below the TFBS track, there is a wiggle-track with base-wise phyloP 100-vertebrate-genomes conservation scores [110, 111]. Further below, there is a track with DNase I hypersensitive areas assayed in 125 ENCODE cell types (which indicate open, transcriptionally-accessible chromatin). The number to the left of each DNase-box indicates the number of cell types in which it was present. The darkness of each box is proportional to the maximal score in any of the cell types. (PDF) [file pone.0208952.s002.pdf]

(A) ROBO2-TSS2

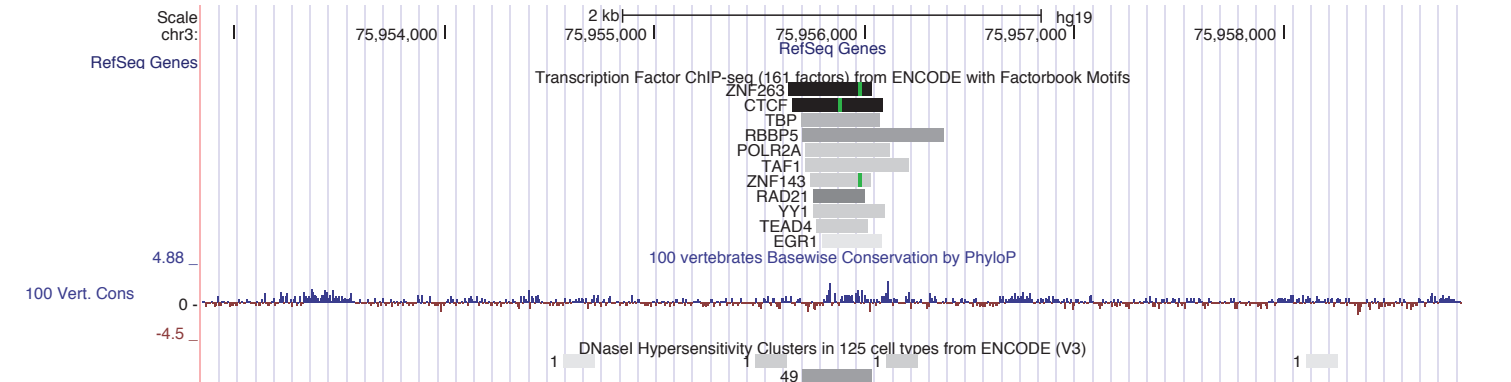

(B) ROBO1-TSS2

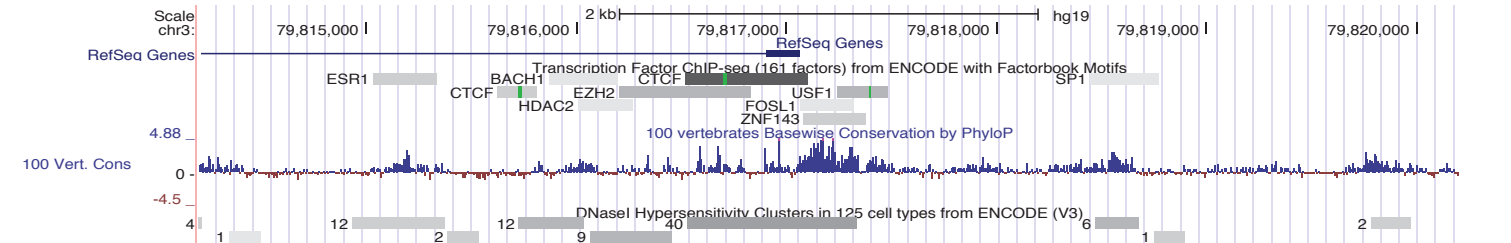

(C) ROBO3-TSS1

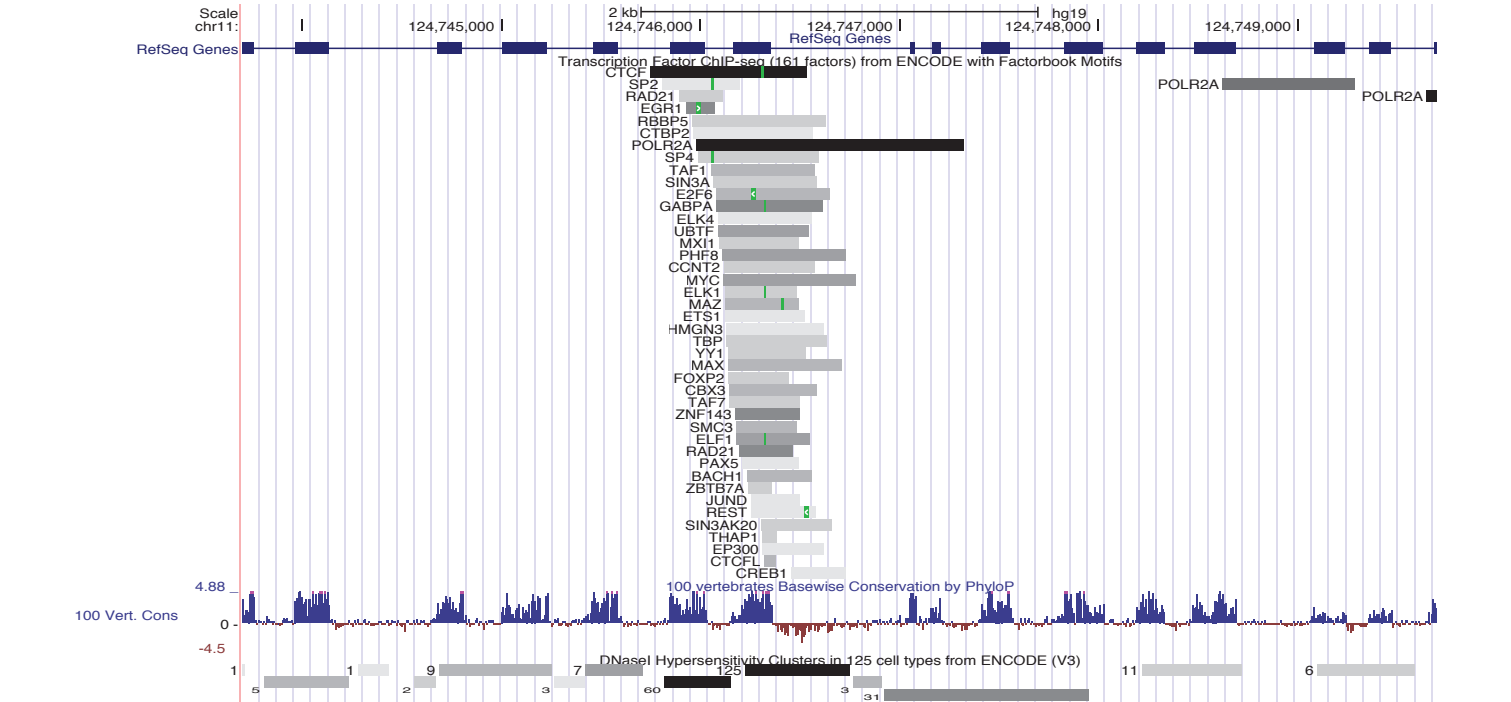

(D) ROBO2-TSS1

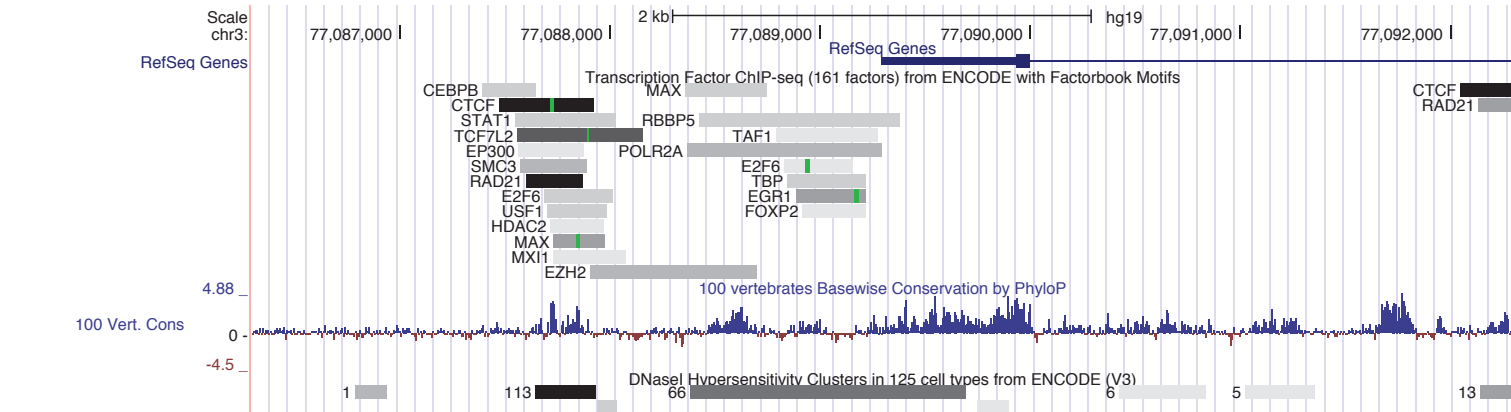

Supplement: S3 Fig — This figure shows additional roundabout promoters shown in the layout analogous to the S2 Fig. (PDF) [file pone.0208952.s003.pdf]
