# Supplementary material for: Magic roundabout is an endothelial-specific ohnolog of ROBO1 which neo-functionalized to an essential new role in angiogenesis
Source: PLoS One. 2019 Feb 25;14(2):e0208952. doi: 10.1371/journal.pone.0208952 (PMC6389290; doi:10.1371/journal.pone.0208952)
Supplement: S4 Fig — The figure shows a phylogenetic tree for a TreeFam family with accession number TF351053. (PDF) [file pone.0208952.s004.pdf]

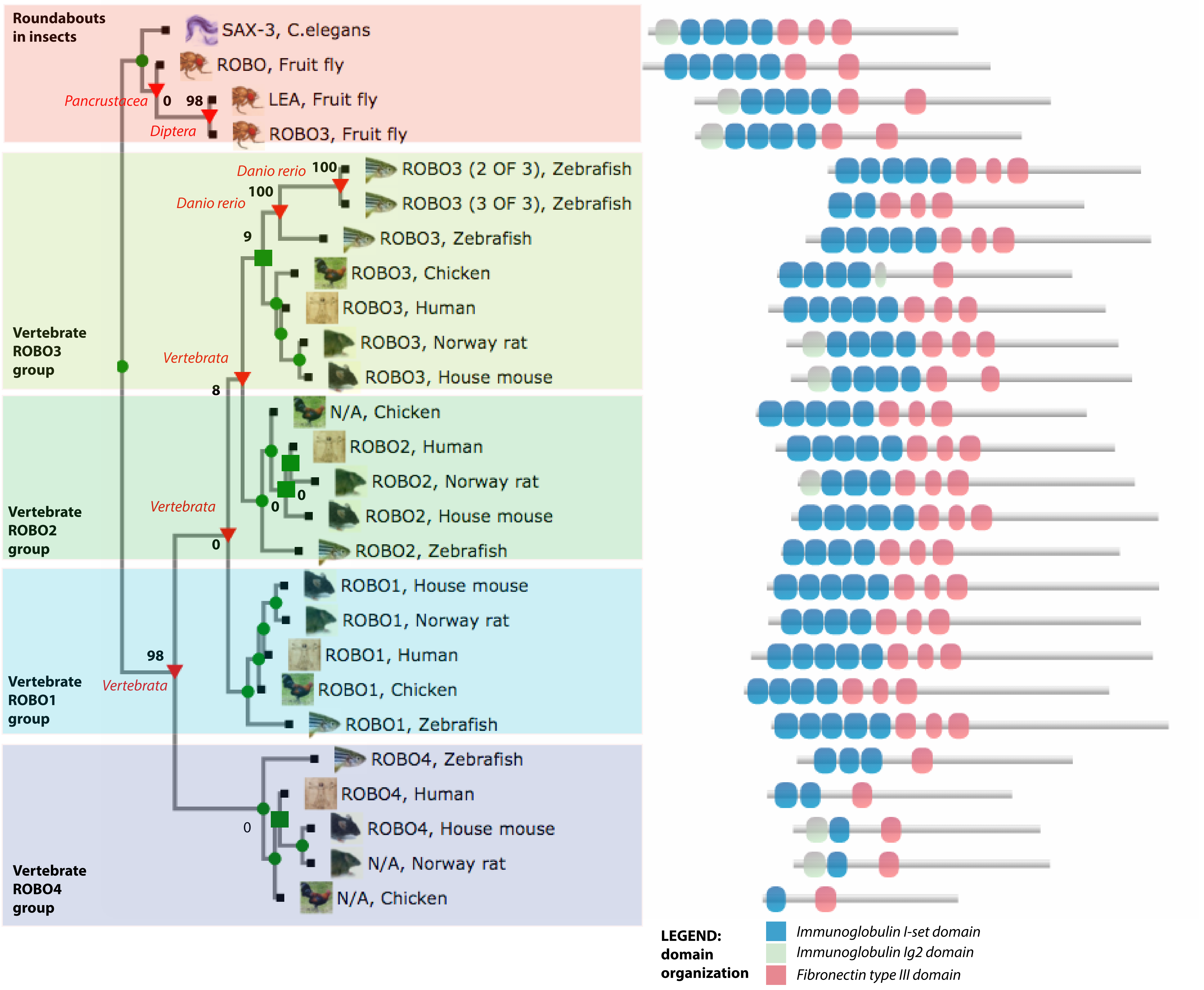

Roundabouts in insects
SAX-3, C.elegans
ROBO, Fruit fly
LEA, Fruit fly
ROBO3, Fruit fly
Pancrustacea
Diptera
Vertebrate ROBO3 group
ROBO3 (2 OF 3), Zebrafish
ROBO3 (3 OF 3), Zebrafish
ROBO3, Zebrafish
ROBO3, Chicken
ROBO3, Human
ROBO3, Norway rat
ROBO3, House mouse
Danio rerio
Vertebrata
Vertebrate ROBO2 group
N/A, Chicken
ROBO2, Human
ROBO2, Norway rat
ROBO2, House mouse
ROBO2, Zebrafish
Vertebrate ROBO1 group
ROBO1, House mouse
ROBO1, Norway rat
ROBO1, Human
ROBO1, Chicken
ROBO1, Zebrafish
Vertebrate ROBO4 group
ROBO4, Zebrafish
ROBO4, Human
ROBO4, House mouse
N/A, Norway rat
N/A, Chicken
LEGEND: domain organization
Immunoglobulin I-set domain
Immunoglobulin Ig2 domain
Fibronectin type III domain
