# Supplementary material for: Magic roundabout is an endothelial-specific ohnolog of ROBO1 which neo-functionalized to an essential new role in angiogenesis
Source: PLoS One. 2019 Feb 25;14(2):e0208952. doi: 10.1371/journal.pone.0208952 (PMC6389290; doi:10.1371/journal.pone.0208952)
Supplement: S1 File — This file contains a number of supplementary results: (1) on the conservation of roundabout clusters, (2) an alternative evolutionary model for the emergence of the roundabout cluster, (3) an analysis of expression patterns in genomic sub-regions neighboring the two roundabout clusters, (4) the simulation tests of the DDC model, and (5) the analysis of promoter probabilities. The file also contains two supplementary tables. The first table is devoted to the analysis of expression profiles in the genomic locus of ROBO4 (Table A). The second table is devoted to the probabilistic analysis of the promoter architectures the TSSes associated with roundabouts (Table B). (DOCX) [file pone.0208952.s005.docx]

**Supplementary Results.**

**The conservation of roundabout clusters.**

Significantly, the ROBO3-ROBO4 cluster is conserved in all vertebrate species for which contiguous chromosomal data were found (Table A). These species were as follows: cat (*Felis catus*), dog (*C. lupus familiaris*), armadillo (*Dasypus novemcinctus*), cow (*Bos taurus*), Western clawed frog (*Xenopus tropicalis*), zebrafish (*Danio rerio*), common bottlenose dolphin (*Tursiops truncatus*), and horse (*Equus caballus*). In many other sequenced vertebrates, genomic data is less reliable, as although some genomes have been sequenced, the assembly of sequences into chromosomes is problematic.

However, caution is necessary when assigning functional significance to the conservation of ROBO3-ROBO4. Such conservation is common. In mammalian evolution, tail-to-tail pairs were found to be as conserved as head-to-head duos ([Franck, Hulsen et al. 2008](#_ENREF_3)), even though the latter are more like to be co-expressed from bi-directional promoters ([Hansen, Bross et al. 2003](#_ENREF_4)). As the orientation of a gene pair does not matter, we ignored it. To estimate the frequency of the conservation of a two-gene human cluster similar to ROBO3-ROBO4 throughput vertebrates, we identified 1051 similar clusters for which one-to-one orthologs were available in all the species of Table A. As many as 323 of the pairs (31%) were conserved in all the seven species. 509 of the pairs (48%) were conserved in at least six. The degree of conservation was the lowest in the frog (76%) and zebrafish (40%); for the remaining species it varied between 96-98%. We note, however, that the commonality of the preservation of such clusters does not preclude the fact that they could be conserved by natural selection.

**An alternative model.**

An alternative evolutionary scenario of the emergence of vertebrate roundabout clusters would be that ROBO3/ROBO4 and ROBO1/ROBO2 are two pairs of tandem duplicates of an ancestrally duplicated gene. The ancestral gene would be duplicated segmentally or through 2R-WGD. Such a scenario is, however, less parsimonious than the *tandem-plus-WGD* model demanding two independent tandem duplications, both followed by inversions. Moreover, the above alternative scenario is not better at explaining the topology of the ROBO4 branch. Yet another alternative is that ROBO1 first tandem duplicated giving rise to ROBO4, then again giving rise to ROBO3, and then again duplicated in tandem giving rise to ROBO2. The entire cluster was then broken into two parts by a chromosomal rearrangement. The last scenario explains the phylogenetic tree (Figure 2), but is in disagreement with the knowledge of the functional preferences of tandem versus WGD duplications. Moreover, it seems unlikely that these tandem duplications would have all occurred at the base of vertebrates, but all the ohnologs were lost. Therefore, on balance, the author is inclined to accept the *tandem-plus-WGD* model and reject the alternative models.

**Expression patterns in genomic sub-regions neighboring the two roundabout clusters.**

As both genes in the ROBO3-ROBO4 cluster have some endothelial expression (ROBO4-TSS1 was enriched in endothelial samples with the z-score of 10), it might be tempting to conclude that the entire ROBO3-ROBO4 cluster is under the influence of an endothelial-specific enhancer. However, only a three kilobase fragment of the ROBO4 proximal promoter was found sufficient to drive endothelial-specific expression in both mice ([Okada, Yano et al. 2007](#_ENREF_5)) and zebrafish ([Samant, Schupp et al. 2011](#_ENREF_6)). Moreover, the expression of ROBO3-TSS1 is not conserved in mouse where there is only one TSS linked with ROBO3 (mostly in the cerebellum, data not shown). Finally, the endothelial signal of ROBO3-TSS1 is two orders of magnitude lower than that of ROBO4-TSS1. Therefore, it is more likely that the weak endothelial expression of ROBO3-TSS1 is a just side-effect of the powerful activation of ROBO4-TSS1 which opens the chromatin in this region. Similar ripple effect that was demonstrated previously for the mitogen-activated protein kinase pathway ([Ebisuya, Yamamoto et al. 2008](#_ENREF_1)).

However, we did test the hypothesis that the endothelial expression of ROBO4 could be coordinated with the expression of neighboring genes. To this purpose, the expression profiles of six sub-regions of the broader ROBO4 locus (Table A) was examined. The sub-regions ranged between 27 and 266 kilobase pairs in size. All CAGE tags mapping in the broad genomic regions were summed over which is a unique mode of analysis possible for F5-CAGE with the Zenbu browser. The results showed that only the sub-region associated with ROBO4 (sub-region IV) had a potent signal derived from ECs. The other sub-regions instead displayed a preference for expression in nervous tissues or the osteosarcoma cell-line ⎯ SAOS-2. Thus the hypothesis of the dominant impact of locus-level regulatory context on the expression pattern of ROBO4 was rejected.

**The simulation tests of the DDC model for ROBO4-TSS1 and ROBO1-TSS1 promoter architectures.**

In the DDC model of paralog evolution, a paralog copy shields its duplicate from purifying selection increasing the chances of the fixation of formerly forbidden deleterious mutations. This ought to lead to the partitioning of regulatory modules between a duplicated pair of genes by degenerative mutations. The resulting paralog copies should share some ancestral regulatory sites preserving others differentially. The proportion of shared sites should reflect the randomness of degenerative mutations.

Two types of simulation models (labeled *independent* and *clustered*) were constructed to prove that a larger ancestral promoter architecture of cardinality equaling 14 could not have given rise to ROBO4-TSS1 (with 7 distinct TFBSes) and ROBO1-TSS1 (with 9 TFBSes) through random TFBS losses. The observed overlap of strong sites between the pair of paralogs was too low (only two TFBSes were in common). In the independent-TF model, TFBSes were modeled as independent sites. In the clustered-TF model, TFBSes were physically clustered in the genome exactly as in the empirically observed architectures of the two roundabout paralogs. The simulations led to the *P*-values of 0.0104 and 0.0406 for independent and clustered models, respectively. Thus, both simulations led to the rejection of the DDC model.

We note that additional processes such as gene conversion would only homogenize paralog promoters increasing the number of shared TFBSes. Moreover, empirical ENCODE data suggest that TFBSes are rarely modular and independent. Instead, they are overlapping and hierarchically embedded. This should also increase the similarity of paralog promoter architectures. This was already noted in Figure 4 of Force *et al.* in their original review introducing the DDC model ([Force, Lynch et al. 1999](#_ENREF_2)).

While the above simulations were only a first attempt to model DDC and a peripheral results, they did add to the evidence in support of the neo-functionalization model for the emergence of ROBO4.

**The analysis of promoter probabilities.**

In the next step, we compared the promoter architecture of ROBO3 not just against the other human roundabout paralogs, but also against all other genes in the genome. Here, the following question was asked: was the architecture of the endothelial-specific promoter of ROBO4 “statistically significant”, or was it a low-complexity arrangement of TFs that could have arisen by chance alone? To answer this question, we compared the observed and expected probabilities of the architectures of roundabouts and other RefSeq genes. The observed probability was simply the empirically observed frequency of a given set of TFs in the set of all proximal promoters in the human genome. The expected probability was the random expectation of the frequency of a given architecture, given the observed frequencies of individual TFs. Enrichment was the ratio of the two probabilities. The higher the enrichment ratio, the less likely that the architecture was due to chance alone^[[1]](#footnote-1)^.

The results are summarized in Table B. The endothelial-specific architecture of the promoter of ROBO4-TSS1 was unlikely to be assembled by chance. No other proximal promoter bound exactly the same set of ENCODE TFs. The neuronal-specific promoter architecture of ROBO1-TSS1 was also a unique and complex architecture. In contrast, the promoter architectures of ROBO2-TSS2, ROBO1-TSS2, ROBO3-TSS1, and ROBO2-TSS1 were low-complexity architectures which might have arisen by chance alone. It can be speculated that such low complexity architectures are also more straightforward to assemble *de novo* and, therefore, more likely to have emerged fortuitously. This is in agreement with the observation that all three TSSes with enrichment > 10,000 were conserved in mouse, while only two of the four low complexity architectures were conserved.

**Table A. The expression profiles of the sub-locations in the ROBO4 genomic locus.**

For each TSS, we show either the expression signal in each individual library (bold font) or enrichment in sets of samples grouped in sample ontologies.

| Sub-locations within the locus  (all positions are on chromosome 11,  in the coordinates of the hg19 assembly) | The top five tissues of expression with signal in in tags per million (TPM);  or z-score of enrichment in a sample category*  (with the number of samples in the category given in curly brackets) | | sense/ anti-sense |
| --- | --- | --- | --- |
| **Sub-region I**  The SPA17/SIAE/TBRG1/PANX3 sub-locus,  150 kb between 124 – 124.58 Mb | **TPM** | **Saos-2 osteosarcoma treated with ascorbic acid and BGP** | **6/784** |
|  |  | **Saos-2 osteosarcoma cell line, untreated control, day 28** | **55/586** |
|  |  | **Saos-2 osteosarcoma treated with ascorbic acid and BGP, day 28** | **1/589** |
|  |  | **Saos-2 osteosarcoma treated with ascorbic acid and BGP, day 14, oil rep 2** | **57/526** |
|  |  | **Saos-2 osteosarcoma treated with ascorbic acid and BGP, day 14, oil rep 1** | **3/527** |
|  | *z-score* | *osteosarcoma cell line, FF_ont:0100407 (59)* | *8/9* |
|  |  | *SAOS-2 cell, FF_ont:0100971 (57)* | *8/12* |
|  |  | *bone cancer cell line, FF_ont:0100385 (61)* | *7/13* |
|  |  | *calcification induced with ascorbate and BPG, FF_ont:0000332 (54)* | *8/12* |
|  |  | *mesenchyme condensation cell, CL:0000335 (67)* | *6/12* |
| **Sub-region II**  The MSANTD2/NRGN/VSIG2/ESAM sub-locus,  120 kb between 124.58 – 124.7 Mb | **TPM** | **putamen** | **0/6332** |
|  |  | **occipital pole** | **0/6247** |
|  |  | **occipital cortex** | **0/5389** |
|  |  | **postcentral gyrus** | **0/4636** |
|  |  | **temporal lobe** | **0/4592** |
|  | *z-score* | *tissue sample, FF_ont:0000004 (198)* | *19/10* |
|  |  | *adult tissue sample, FF_ont:0000998 (151)* | *16/9* |
|  |  | *endothelial cell, CL:0000115 (83)* | *13/5* |
|  |  | *endothelial cell of vascular tree, CL:0002139 (69)* | *13/5* |
|  |  | *meso-epithelial cell, CL:0002078 (93)* | *13/4* |
| **Sub-region III**  The ROBO3 sub-locus,  53 kb between 124.7 – 124.753 Mb | **TPM** | **endometrial stromal sarcoma cell line** | **0/47** |
|  |  | **H9 Embryoid body cells, melanocytic induction, day 12** | **0/37** |
|  |  | **epithelioid sarcoma cell line** | **0/37** |
|  |  | **nasal epithelial cells** | **0/35** |
|  |  | **small airway epithelial cells** | **0/35** |
|  | *z-score* | *cell by lineage, CL:0000220 (365)* | *0/12* |
|  |  | *ectodermal cell, CL:0000221 (170)* | *0/11* |
|  |  | *embryonic cell, CL:0002321 (457)* | *0/11* |
|  |  | *neurectodermal cell, CL:0000133 (155)* | *0/11* |
|  |  | *ecto-epithelial cell, CL:0002077 (80)* | *0/10* |
| **Sub-region IV**  The ROBO4 sub-locus,  27 kb between 124.753 – 124.78 Mb | **TPM** | **renal glomerular endothelial cells** | **989/0** |
|  |  | **endothelial cells – microvascular, donor 3** | **741/0** |
|  |  | **endothelial cells – microvascular, donor 1** | **612/0** |
|  |  | **endothelial cells – aortic** | **585/0** |
|  |  | **renal glomerular endothelial cells** | **547/0** |
|  | *z-score* | *endothelial cell of vascular tree, CL:0002139 (69)* | *14/8* |
|  |  | *endothelial cell, CL:0000115 (83)* | *14/7* |
|  |  | *meso-epithelial cell (CL:0002078) (93)* | *13/7* |
|  |  | *endothelial cell of lymphatic vessel (CL:0002138) (51)* | *12/7* |
|  |  | *VEGF-C treatment (FF_ont:0000341) (48)* | *12/6* |
| **Sub-region V**  The HEPN/HEPACAM/CCDC15/SLC/TMEM218 sub-locus,  220 kb between 124.78 – 125 Mb | **TPM** | **medial temporal gyrus, adult** | **3576/0** |
|  |  | **substantia nigra – adult** | **2982/0** |
|  |  | **corpus callosum, adult** | **689/0** |
|  |  | **thalamus, adult** | **9/0** |
|  |  | **hippocampus, adult** | **5/0** |
|  | *z-score* | *leukocyte, CL:0000738 (266)* | *0/15* |
|  |  | *myeloid leukocyte, CL:0000766 (180)* | *-2/16* |
|  |  | *nongranular leukocyte, CL:0002087 (206)* | *0/14* |
|  |  | *monocyte, CL:0000576 (135)* | *-2/15* |
|  |  | *monocyte, CL:0000576 (135)* | *0/13* |
| **Sub-region VI**  PKNOX2 sub-locus,  266 kb between 125 – 125.266 Mb | **TPM** | **myoblast differentiation to myotubes, day04, Duchenne dystrophy** | **0/163** |
|  |  | **medial frontal gyrus, newborn** | **0/91** |
|  |  | **occipital cortex, newborn** | **0/80** |
|  |  | **myoblast differentiation to myotubes, day06, Duchenne dystrophy** | **0/79** |
|  |  | **rhabdomyosarcoma cell line** | **0/76** |
|  | *z-score* | *neurectodermal cell, CL:0000133 (155 exps)* | *3/12* |
|  |  | *ectodermal cell, CL:0000221 (170 exps)* | *3/11* |
|  |  | *electrically signaling cell, CL:0000404 (51 exps)* | *4/8* |
|  |  | *iPS differentiation to neuron, FF_ont:0200005 (48 exps)* | *4/8* |
|  |  | *neural cell, CL:0002319 (76 exps)* | *3/8* |

* Groupings of samples derive from sample ontologies: UBERON ([Mungall, Torniai et al. 2012](#_ENREF_54)), or Gene Ontology ([Ashburner, Ball et al. 2000](#_ENREF_1)). Ontologies are sorted according to the Wilcoxon-mann-whitney rank-sum enrichment *z*-score. The top five enriched sample categories are given for each TSS.

Note: locations are given in the coordinates of the hg19 assembly.

**Table B. Probabilities of promoter architectures associated with roundabout TSSes.**

Promoter architectures were sorted according to the degree of enrichment: the first row is the architecture which is least likely to have occurred by chance alone. Note that all roundabout TSSes with the enrichment > 10,000 are conserved in mouse.

| Promoter architecture | Observed probability | | | Expected probability | | Enrichment | Conserved in mouse? |
| --- | --- | --- | --- | --- | --- | --- | --- |
|  | O-value | Perce-ntile | Count | E-value | Perce-ntile |  |  |
| ROBO1-TSS1  **(BHLHE40, CEBPB, CHD1, CTCF, EP300, JUND, MAFK, TAF1,**  **TBP)** | 2e-05 | 0 | 1 | 0 | 0 | 7,047,610 | YES |
| ROBO4-TSS1  **(EBPB, CTCF, FOS, GATA2, JUN, REST, STAT3)** | 2e-05 | 0 | 1 | 0 | 0 | 794,581 | YES |
| ROBO3-TSS2  **(CTCF, E2F6, EZH2, MAX, RAD21, REST, USF1)** | 5e-05 | 32 | 2 | 7e-10 | 41 | 66,820 | YES |
| ROBO2-TSS2  **(CTCF, RAD21, ZNF263)** | 0.00081 | 62 | 33 | 2.1014e-06 | 60 | 384 | no |
| ROBO1-TSS2  **(CTCF)** | 0.0138 | 71 | 564 | 6.35626e-05 | 74 | 217 | YES |
| ROBO3-TSS1  **(CTCF, EGR1, GABPA, RAD21, ZNF143)** | 2e-05 | 0 | 1 | 1.158e-07 | 51 | 211 | no |
| ROBO2-TSS1  **(CTCF, RAD21, TCF7L2)** | 0.00012 | 46 | 5 | 1.5053e-06 | 58 | 81 | YES |

Note: O-value stands for observed probability while E-value stands for expected probability. (O-values were rounded to 5 digits after the coma and E-values to 10 digits). Percentiles are calculated in respect to the distributions of O- and E-values obtained for all human RefSeqs. Enrichment ratio is the ratio of O-value over E-value: indicating enrichment in observed data (human genome) over random expectation.

**Supplementary References.**

Ebisuya, M., T. Yamamoto, et al. (2008). "Ripples from neighbouring transcription." Nature cell biology **10**(9): 1106-1113.

Force, A., M. Lynch, et al. (1999). "Preservation of duplicate genes by complementary, degenerative mutations." Genetics **151**(4): 1531-1545.

Franck, E., T. Hulsen, et al. (2008). "Evolution of closely linked gene pairs in vertebrate genomes." Molecular biology and evolution **25**(9): 1909-1921.

Hansen, J. J., P. Bross, et al. (2003). "Genomic structure of the human mitochondrial chaperonin genes: HSP60 and HSP10 are localised head to head on chromosome 2 separated by a bidirectional promoter." Human genetics **112**(1): 71-77.

Okada, Y., K. Yano, et al. (2007). "A three-kilobase fragment of the human Robo4 promoter directs cell type-specific expression in endothelium." Circulation research **100**(12): 1712-1722.

Samant, G. V., M. O. Schupp, et al. (2011). "Sox factors transcriptionally regulate ROBO4 gene expression in developing vasculature in zebrafish." The Journal of biological chemistry **286**(35): 30740-30747.

1. It is noted that this test is somewhat similar to a Bayes factor (BF). However, the BF lower than one is interpreted as the evidence for the null hypothesis (in this case: randomness). Herein, the enrichment ratio lower than one would instead indicate the observed frequency of an architecture is lower than random expectation. [↑](#footnote-ref-1)
