# Supplementary material for: Magic roundabout is an endothelial-specific ohnolog of ROBO1 which neo-functionalized to an essential new role in angiogenesis
Source: PLoS One. 2019 Feb 25;14(2):e0208952. doi: 10.1371/journal.pone.0208952 (PMC6389290; doi:10.1371/journal.pone.0208952)
Supplement: S5 File — This file contains Mr. Bayes consensus trees with varying analysis parameters. (DOCX) [file pone.0208952.s009.docx]

**The list of MrBayes trees:**

**(1) Protein region = W, rates = equal:**

(dmRobo1:0.674006,hsROBO4:0.645658,(hsROBO3:0.487292,(hsROBO1:0.306459,hsROBO2:0.268801)1.00:0.140769)1.00:0.221615);

Tree with node numbers:

,==============3 dmRobo1

|

=====2|==============4 hsROBO4

|

| ,======6 hsROBO3

`======5|

| ,==8 hsROBO1

`==7|

`==9 hsROBO2

**(2) Protein region = E, rates = equal:**
(dmRobo1:0.564839,hsROBO4:0.508348,(hsROBO3:0.37071,(hsROBO1:0.256365,hsROBO2:0.203211)1.00:0.117815)1.00:0.240686);

Tree with node numbers:

,==============3 dmRobo1

|

=====2|==============4 hsROBO4

|

| ,======6 hsROBO3

`======5|

| ,==8 hsROBO1

`==7|

`==9 hsROBO2

**(3) Protein region = I, rates = equal:**

(dmRobo1:0.934654,hsROBO4:0.73477,(hsROBO3:0.839719,(hsROBO1:0.38528,hsROBO2:0.466449)0.99:0.214225)0.95:0.176344);

Tree with node numbers:

,==============3 dmRobo1

|

=====2|==============4 hsROBO4

|

| ,======6 hsROBO3

`======5|

| ,==8 hsROBO1

`==7|

`==9 hsROBO2

**(4) Protein region = W, rates = gamma:**

(dmRobo1:0.838643,hsROBO4:0.732023,(hsROBO3:0.557288,(hsROBO1:0.336049,hsROBO2:0.284396)0.99:0.153624)1.00:0.281576);

Tree with node numbers:

,==============3 dmRobo1

|

=====2|==============4 hsROBO4

|

| ,======6 hsROBO3

`======5|

| ,==8 hsROBO1

`==7|

`==9 hsROBO2

**(5) Protein region = E, rates = gamma:**

(dmRobo1:0.732298,hsROBO4:0.595714,(hsROBO3:0.424766,(hsROBO1:0.283153,hsROBO2:0.209639)1.00:0.122332)1.00:0.318474);

Tree with node numbers:

,==============3 dmRobo1

|

=====2|==============4 hsROBO4

|

| ,======6 hsROBO3

`======5|

| ,==8 hsROBO1

`==7|

`==9 hsROBO2

**(6) Protein region = I, rates = gamma:**

(dmRobo1:0.962095,hsROBO4:0.757845,(hsROBO3:0.849458,(hsROBO1:0.392107,hsROBO2:0.470744)1.00:0.207795)0.95:0.187879);

Tree with node numbers:

,==============3 dmRobo1

|

=====2|==============4 hsROBO4

|

| ,======6 hsROBO3

`======5|

| ,==8 hsROBO1

`==7|

`==9 hsROBO2

**(7) Protein region = W, rates = invgamma:**

(dmRobo1:0.795312,hsROBO4:0.678288,(hsROBO3:0.533092,(hsROBO1:0.321431,hsROBO2:0.279068)1.00:0.138753)0.99:0.253938);

Tree with node numbers:

,==============3 dmRobo1

|

=====2|==============4 hsROBO4

|

| ,======6 hsROBO3

`======5|

| ,==8 hsROBO1

`==7|

`==9 hsROBO2

**(8) Protein region = E, rates = invgamma:**

(dmRobo1:0.672319,hsROBO4:0.554306,(hsROBO3:0.402153,(hsROBO1:0.268987,hsROBO2:0.2057)0.99:0.114153)1.00:0.279274);

Tree with node numbers:

,==============3 dmRobo1

|

=====2|==============4 hsROBO4

|

| ,======6 hsROBO3

`======5|

| ,==8 hsROBO1

`==7|

`==9 hsROBO2

**(9) Protein region = I, rates = invgamma:**

(dmRobo1:0.952568,hsROBO4:0.766838,(hsROBO3:0.855578,(hsROBO1:0.391924,hsROBO2:0.469315)0.99:0.208681)0.96:0.19255);

Tree with node numbers:

,==============3 dmRobo1

|

=====2|==============4 hsROBO4

|

| ,======6 hsROBO3

`======5|

| ,==8 hsROBO1

`==7|

`==9 hsROBO2
